# Molecular Docking of Natural Compounds as DPP-4 Inhibitors in Type 2 Diabetes: A Comprehensive Review

**DOI:** 10.3390/pharmaceutics18060741

**Published:** 2026-06-15

**Authors:** Justyna Baranowska, Anna Kiss, Łukasz Szeleszczuk

**Affiliations:** 1 Department of Organic and Physical Chemistry, Faculty of Pharmacy, Medical University of Warsaw, Banacha 1 Str., 02-093 Warsaw, Poland; justyna.baranowska@wum.edu.pl; 2 Chair and Department of Pharmaceutical Biology, Medical University of Warsaw, Banacha 1, 02-097 Warsaw, Poland; anna.kiss@wum.edu.pl; 3 Doctoral School, Medical University of Warsaw, Żwirki i Wigury 81 Str., 02-093 Warsaw, Poland

**Keywords:** DPP-4, type 2 diabetes mellitus, molecular docking, natural compounds, molecular dynamics, virtual screening, drug discovery

## Abstract

Dipeptidyl peptidase-4 (DPP-4) is an established therapeutic target in the treatment of type 2 diabetes mellitus (T2DM), primarily due to its role in regulating incretin activity and glucose homeostasis. Although clinically approved DPP-4 inhibitors are widely used, their moderate efficacy has driven the search for novel compounds with improved properties. In this context, natural products have attracted considerable attention as a source of structurally diverse and biologically active molecules. At the same time, molecular docking has emerged as a key computational tool for the identification and evaluation of potential DPP-4 inhibitors. This review summarizes and critically analyzes current molecular docking studies of natural compounds targeting DPP-4. Over 150 studies were evaluated with respect to docking methodologies, selection of protein structures, and validation strategies. The results reveal substantial variability in computational protocols. Frequently used protein structures include ligand-bound DPP-4 models such as 1X70 and 6B1E. Among the investigated compounds, flavonoids represent the most extensively studied class, followed by alkaloids, phenolics, terpenoids, and peptides. Despite numerous reports of favorable binding interactions within the DPP-4 active site, many studies rely solely on docking results without further validation. The limited use of molecular dynamics simulations and experimental assays highlights a significant gap in the current literature. Overall, while molecular docking provides valuable preliminary insights, improved standardization and integration with complementary approaches are essential to enhance the reliability and translational relevance of in silico findings.

## 1. Introduction

Diabetes mellitus is a chronic metabolic disorder that represents a major global health problem, with a continuously increasing prevalence worldwide. Type 2 diabetes (T2DM), which accounts for the vast majority of cases, is characterized by impaired glucose homeostasis caused by insulin resistance or inadequate insulin production. Poor glycemic control is associated with the development of long-term complications, including cardiovascular disease, neuropathy, nephropathy, and retinopathy. This highlights the importance of the development of more effective therapeutic strategies aimed at maintaining stable blood glucose levels [1,2].

Among the various molecular targets explored in antidiabetic therapy, dipeptidyl peptidase-4 (DPP-4) has gained particular attention due to its key role in regulating incretin hormone activity. The development of DPP-4 inhibitors, commonly known as “gliptins”, has provided a clinically relevant approach to improving glycemic control through enhancement of endogenous incretin effects. However, despite their clinical success, these agents exhibit moderate efficacy, which continues to drive the search for novel inhibitors with improved properties [3].

In this context, natural compounds have emerged as promising candidates due to their structural diversity and wide range of biological activities. At the same time, advances in computational methods have significantly accelerated early-stage drug discovery. In particular, molecular docking has become a widely used approach for predicting ligand-protein interactions and identifying potential inhibitors prior to experimental validation.

Despite the growing number of docking studies on natural DPP-4 inhibitors, significant inconsistencies in computational methodologies, protein selection, and validation strategies limit the comparability and translational relevance of reported findings.

The aim of this review is to summarize current knowledge on DPP-4 as a therapeutic target and to critically analyze the potential of natural compounds as DPP-4 inhibitors, with a particular focus on studies employing molecular docking approaches.

## 2. Literature Search Strategy and Scope of the Review

The present article was designed as a comprehensive scoping review aimed at providing an overview of molecular docking studies investigating natural compounds as potential DPP-4 inhibitors, rather than as a formal systematic review. Accordingly, the objective was not to perform a quantitative meta-analysis or quality scoring of individual studies, but rather to identify major methodological trends, summarize the diversity of investigated compounds, and critically discuss the strengths and limitations of current computational approaches.

The literature survey was conducted using three major databases, namely Scopus, Web of Science, and PubMed. Searches were performed up to March 2026. The primary search terms included “DPP-4 docking” and “DPP-IV docking”, which were supplemented by combinations with terms such as “natural compounds”, “phytochemicals”, “flavonoids”, “alkaloids”, “phenolics”, “terpenoids”, “peptides”, and “molecular docking”. Additional articles were identified through manual examination of references cited in relevant publications.

After removal of duplicate records, titles and abstracts were screened to identify studies investigating natural compounds as potential DPP-4 inhibitors using molecular docking approaches. Only original research articles published in peer-reviewed journals and written in English were considered. Studies focused exclusively on synthetic compounds, review articles, conference abstracts, editorials, and papers lacking docking analyses were excluded. In addition to plant-derived metabolites, studies involving food-derived bioactive peptides were also included because these compounds represent an important group of naturally occurring molecules with potential DPP-4 inhibitory activity.

Subsequently, full-text articles were evaluated for relevance and suitability. A total of 178 studies met the inclusion criteria and were subjected to detailed analysis. Information extracted from each study included the investigated compound(s), docking software employed, protein structures used, availability of molecular dynamics simulations, and the presence or absence of experimental validation.

Given the substantial methodological heterogeneity among the available reports, no formal quality-assessment framework or meta-analytical procedures were applied. Instead, emphasis was placed on identifying common trends, frequently used computational protocols, and major limitations affecting the reliability and translational value of docking-based studies.

The resulting dataset served as the basis for the qualitative and semi-quantitative analyses presented throughout this review.

## 3. DPP-4 as a Therapeutic Target in Diabetes

### 3.1. Role of DPP-4 in Glucose Metabolism

Dipeptidyl peptidase-4 is a widely expressed serine protease that plays a central role in the regulation of glucose homeostasis through modulation of incretin hormone activity [3,4]. It occurs both as a membrane-bound enzyme and in a soluble circulating form, reflecting its involvement in systemic metabolic regulation.

A key physiological function of DPP-4 is the rapid inactivation of incretin hormones, primarily glucagon-like peptide-1 (GLP-1) and glucose-dependent insulinotropic polypeptide (GIP), which are secreted from the gastrointestinal tract in response to nutrient intake [3,5]. These hormones contribute to postprandial glucose control by stimulating insulin secretion and suppressing glucagon release in a glucose-dependent manner. In addition, GLP-1 exerts extra-pancreatic effects, including slowing gastric emptying and reducing hepatic glucose production [3].

DPP-4 enzymatically cleaves incretins at the N-terminal region, particularly in peptides containing proline or alanine at the penultimate position, leading to rapid loss of their biological activity [4,5]. As a consequence, active GLP-1 has an extremely short half-life in circulation, typically around 1–2 min, which significantly limits the duration of its physiological effects [3,5]. Through this rapid degradation, DPP-4 tightly regulates incretin bioavailability and ensures precise temporal control of glucose-lowering signals.

**Figure 1:**
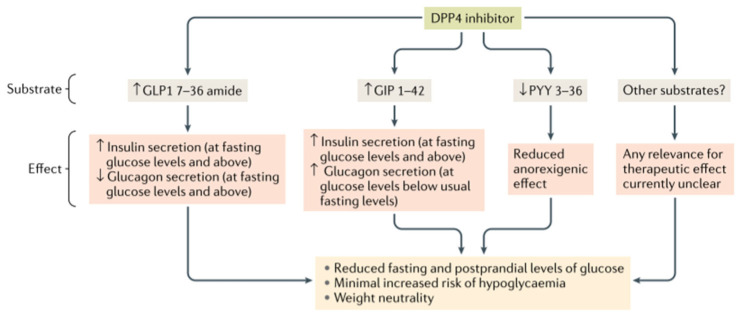
Mechanism of action of DPP-4 inhibitors. Adapted with permission from [3].

Overall, DPP-4 acts as a key enzymatic checkpoint in glucose metabolism, controlling the intensity and duration of incretin-mediated effects on insulin secretion and glucagon suppression. This regulatory role provides the biological rationale for targeting DPP-4 in the treatment of type 2 diabetes mellitus (Figure 1).

### 3.2. Clinically Used DPP-4 Inhibitors

Given the central role of DPP-4 in incretin inactivation, pharmacological inhibition of this enzyme has become an established strategy in the management of type 2 diabetes mellitus. DPP-4 inhibitors, commonly referred to as “gliptins”, include several clinically approved agents such as sitagliptin, vildagliptin, saxagliptin, alogliptin, linagliptin, teneligliptin, omarigliptin, and trelagliptin [4,6]. These compounds differ in their chemical structures and pharmacokinetic profiles but share a common mechanism of action.

DPP-4 inhibitors exert their therapeutic effect by preventing the enzymatic degradation of endogenous incretins, thereby prolonging their activity and enhancing physiological glucose regulation. This leads to increased insulin secretion and reduced glucagon levels in a glucose-dependent manner, which minimizes the risk of hypoglycemia compared to traditional insulin secretagogues [3].

In clinical settings, DPP-4 inhibitors are used both as monotherapy and in combination with other antidiabetic agents, particularly metformin. They have been shown to provide moderate but consistent reductions in glycated hemoglobin (HbA1c) and improvements in both fasting and postprandial glucose levels [3,4]. An additional advantage of this drug class is its neutral effect on body weight and generally favorable tolerability profile, making it suitable for a wide range of patients.

However, despite these benefits, DPP-4 inhibitors exhibit lower glucose-lowering efficacy compared to some newer therapeutic options, especially in patients with advanced T2DM and reduced β-cell function [3]. Furthermore, although these agents are considered safe, rare adverse events such as pancreatitis have been reported [3,7]. Differences in pharmacokinetic properties among individual drugs may also influence dosing frequency and clinical use.

**Figure 2:**
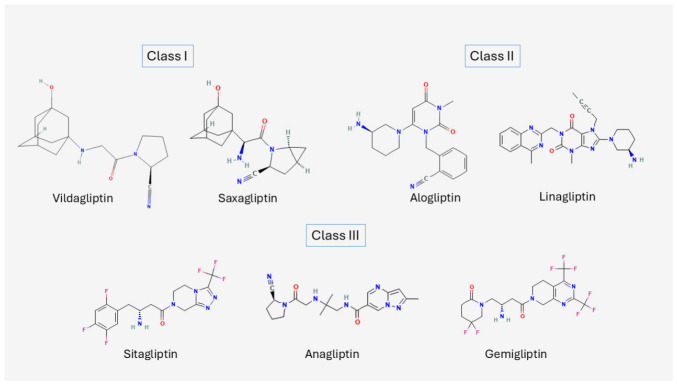
Examples of approved DPP-4 inhibitors.

Taken together, DPP-4 inhibitors are a well-tolerated therapeutic class targeting incretin metabolism (Figure 2). However, their moderate efficacy drives the search for novel agents with improved potency. To further improve the efficacy and selectivity of DPP-4 inhibitors, a detailed understanding of the structural features of the enzyme and its active site is required.

### 3.3. Structural Features of the DPP-4 Active Site

Dipeptidyl peptidase-4 is a membrane-associated serine protease that functions as a homodimer, with each monomer composed of a catalytic domain and an eight-bladed β-propeller domain [4]. The active site is located within the extracellular region of the enzyme, forming a relatively large binding cavity capable of accommodating structurally diverse ligands [6]. However, substrate recognition and inhibitor specificity are primarily governed by well-defined subpockets within the active site, including the S1, S2, and S2 extensive regions, as well as the catalytic triad (Ser630, Asp708, His740), which collectively determine binding orientation and interaction patterns.

**Figure 3:**
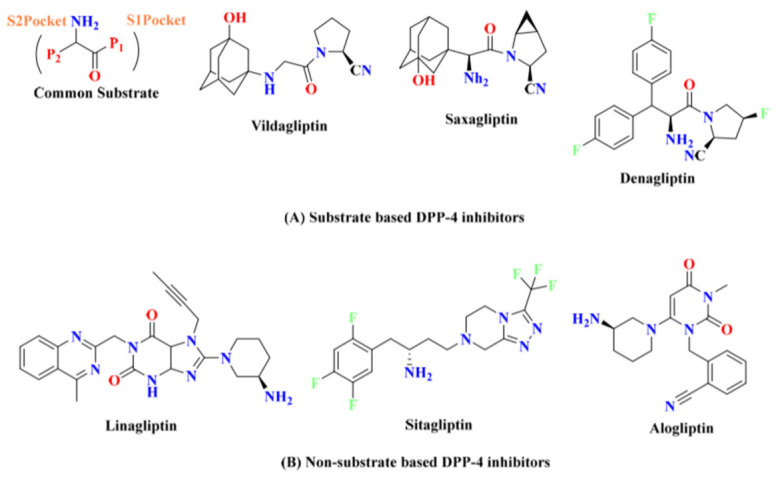
Substrate and non-substrate-based DPP-4 market approved inhibitors. Adapted with permission from [4].

The architecture of the active site is defined by a series of interconnected subsites, including S1, S2, S1′, S2′, and the S2 extensive region, which together create a complex and versatile binding environment [6]. Among these, the S1 and S2 pockets play a central role in ligand recognition and are essential for inhibitory activity, whereas additional interactions within the S1′, S2′, or S2 extensive regions can significantly enhance binding affinity and potency [4]. Functionally, the S1 pocket is predominantly hydrophobic and accommodates nonpolar fragments of ligands, while the S2 subsite provides a more polar environment that enables hydrogen bonding and electrostatic interactions, thereby contributing to ligand stabilization (Figure 3) [4,6].

At the molecular level, ligand binding within the DPP-4 active site is governed by a network of conserved amino acid residues that ensure both specificity and stability of the complex. In particular, Glu205 and Glu206 play a pivotal role in anchoring ligands through hydrogen bonding interactions, forming a key recognition motif within the catalytic pocket [6]. Additional residues, such as Tyr662 and Asn710, contribute to the precise orientation and stabilization of bound ligands, while Arg125 is involved in positioning specific functional groups within the binding cavity, supporting a conserved binding mode across structurally diverse inhibitors [6,8].

Despite the structural diversity of DPP-4 inhibitors, crystallographic studies consistently demonstrate that the overall architecture of the active site remains largely preserved upon ligand binding, with only minor adjustments observed in selected side chains [6,8]. This relative rigidity suggests that ligand recognition in DPP-4 relies more on precise complementarity to a preorganized binding pocket than on large-scale conformational changes. Notably, conserved water molecules within the active site have been shown to play a critical role in maintaining the orientation of key residues, particularly Glu205 and Glu206, and in facilitating proper ligand positioning within the S2 subsite [6].

From a molecular docking perspective, these structural characteristics are of fundamental importance. The presence of multiple subsites enables ligands to adopt diverse binding modes, which must be accurately captured during computational modeling. At the same time, the conserved arrangement of key residues and the involvement of water-mediated interactions highlight the need to account for both direct protein-ligand contacts and solvent effects in docking studies [8]. Furthermore, the relatively large and adaptable binding cavity of DPP-4 necessitates careful conformational sampling and thorough validation of predicted binding poses to ensure reliable results. These features are particularly relevant in the context of natural compounds, which exhibit high structural diversity and complexity, making molecular docking an essential tool for exploring their potential as DPP-4 inhibitors.

## 4. Computational Approaches in DPP-4 Inhibitor Research

### 4.1. Why Molecular Docking Is Widely Used?

Computational approaches have become an integral part of modern drug discovery, particularly at the early stages of identifying potential bioactive compounds. Among these methods, molecular docking is one of the most widely applied techniques for evaluating ligand-protein interactions, enabling rapid screening of large compound libraries and prediction of potential binding modes within a target protein.

One of the key advantages of molecular docking is its ability to provide a cost-effective and time-efficient strategy for prioritizing candidate molecules prior to experimental validation. By estimating binding affinity and identifying key interactions within the active site, docking studies allow researchers to narrow down large sets of compounds and focus on the most promising candidates. This is particularly relevant in the context of natural products, which are characterized by high structural diversity and often require extensive screening to identify biologically active compounds.

**Figure 4:**
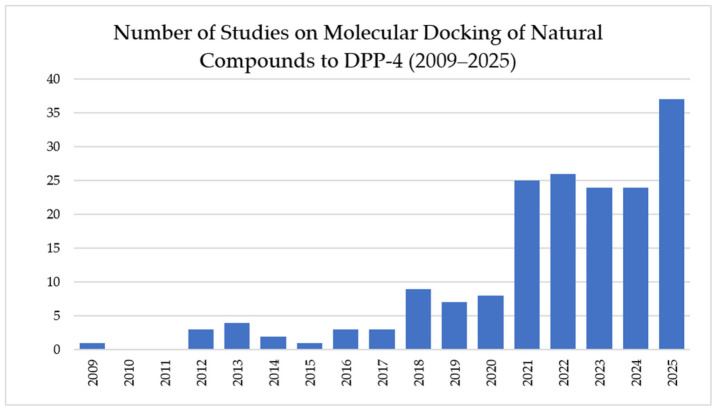
Number of publications per year reporting molecular docking studies of natural compounds targeting DPP-4. The analysis was based on literature retrieved from the Scopus, Web of Science, and PubMed databases. Only original research articles investigating natural compounds using molecular docking approaches were included.

In recent years, there has been a noticeable increase in the number of studies employing molecular docking to investigate natural compounds as potential DPP-4 inhibitors. This growing trend reflects both the expanding interest in natural product-based drug discovery and the accessibility of docking software. The increasing number of publications in this field is illustrated in Figure 4, which presents the rising trend of docking studies over time.

### 4.2. General Workflow of Molecular Docking Studies

Molecular docking studies typically follow a multistep workflow that includes protein preparation, ligand preparation, definition of the binding site, docking calculations, and analysis of the resulting ligand-protein interactions. Although these steps are conceptually straightforward, their proper execution is critical for obtaining reliable and reproducible results [9].

A key aspect of any docking study is the selection of an appropriate protein structure. High-resolution crystal structures are generally preferred, as they provide more accurate atomic coordinates within the binding site. In addition, the presence of a co-crystallized ligand is highly advantageous, as it facilitates precise identification of the active site and enables validation of the docking protocol through re-docking approaches.

Equally important is the choice of docking software, as different programs employ distinct search algorithms and scoring functions, which may lead to variations in predicted binding modes and affinity estimates. Consequently, the selection of the docking platform and parameter settings can significantly influence the outcome of the study [10].

Ligand preparation represents another critical step, particularly in the case of natural compounds, which often exhibit structural complexity, multiple ionization states, and conformational flexibility. Inadequate ligand preparation may lead to unrealistic binding poses or misleading docking scores.

Following docking calculations, analysis of binding modes typically focuses on identifying key interactions within the active site, including hydrogen bonds, hydrophobic contacts, and π–π interactions with residues involved in ligand recognition.

Importantly, molecular docking provides a static representation of ligand-protein interactions. Therefore, increasing attention is being paid to the integration of molecular dynamics (MD) simulations, which allow evaluation of the stability of the docked complex over time and provide insight into protein flexibility and solvent effects. The inclusion of MD simulations can significantly improve the reliability of in silico predictions and should be considered an important component of a comprehensive computational workflow [11,12].

### 4.3. Docking Software Used in Studies of Natural DPP-4 Inhibitors

**Figure 5:**
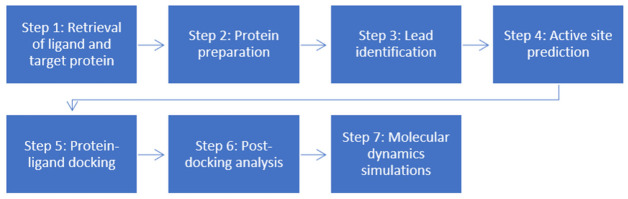
Flow chart of a molecular docking study.

To better understand current computational practices in studies investigating natural inhibitors of DPP-4, the docking software used in the reviewed literature was analyzed. The results of this analysis indicate a clear dominance of AutoDock-based platforms in molecular docking studies investigating natural compounds as potential inhibitors of DPP-4. Among the evaluated software tools, AutoDock Vina was the most frequently used program (56 studies), followed by AutoDock (32 studies). Together, these tools accounted for the majority of docking analyses reported in the literature, highlighting their popularity in studies involving natural products. Their widespread application is likely related to their open accessibility, ease of use, and extensive validation in protein-ligand interaction studies (Figure 5).

Other docking platforms were used less frequently. Glide was the third most commonly employed software (18 studies), reflecting its established role as a high-accuracy docking tool within commercial computational chemistry suites. Molegro Virtual Docker (10 studies) and Molecular Operating Environment (MOE) (6 studies) were also applied in several investigations, whereas programs such as CDOCKER, Discovery Studio, SYBYL, GOLD, FRED, and FlexX appeared only sporadically.

In addition, a small number of studies utilized web-based docking servers, including CB-Dock and DockingServer.com. Although such tools offer convenient access to docking workflows, their limited use suggests that most researchers prefer standalone docking software that provides greater control over docking parameters and scoring functions.

Overall, the results demonstrate that molecular docking studies of natural DPP-4 inhibitors are strongly concentrated around a limited number of widely used platforms, particularly AutoDock Vina and AutoDock (Table 1). However, the diversity of applied software indicates considerable methodological heterogeneity across studies. Differences in docking algorithms, scoring functions, and protein preparation protocols may significantly influence predicted binding affinities and interaction patterns. For example, AutoDock-based methods rely on empirical free-energy scoring functions, whereas programs such as Glide or MOE employ different scoring schemes and search algorithms. These methodological differences may lead to variations in predicted binding modes and docking scores across studies and should therefore be considered when comparing results reported in independent investigations.

Despite the widespread application of molecular docking in the analyzed studies, only a limited number of investigations further validated the predicted protein-ligand complexes using molecular dynamics simulations. This indicates that the majority of computational studies on natural DPP-4 inhibitors rely primarily on docking results alone.

While docking is a valuable tool for predicting potential binding modes and estimating binding affinity, it represents a static approximation of protein-ligand interactions. In contrast, MD simulations allow the evaluation of the stability of the ligand within the binding pocket over time, taking into account protein flexibility, solvent effects, and conformational changes in the complex. Therefore, the limited use of MD simulations represents a methodological gap in the current literature. Future computational investigations of natural DPP-4 inhibitors would benefit from integrating docking with MD simulations to improve the reliability of in silico predictions.

### 4.4. Protein Structures Used in Docking Studies

To further characterize the structural models used in docking studies of natural DPP-4 inhibitors, the Protein Data Bank (PDB) structures employed across the reviewed literature were analyzed. The results revealed that a relatively small number of DPP-4 crystal structures were repeatedly used as docking templates. Among them, the human DPP-4 structure 1X70, co-crystallized with sitagliptin, was the most frequently applied model (24 studies). Other commonly used structures included 6B1E (vildagliptin-bound, 14 studies), 4A5S containing a synthetic DPP-4 inhibitor (PDB ligand N7F, 12 studies), 2ONC representing an apo structure (11 studies), and 1WCY co-crystallized with Diprotin A (10 studies). Several additional structures, such as 3G0B, 4PNZ, 5T4B, 3F8S, and 2P8S, were used in a moderate number of studies (Table 2).

Importantly, the majority of selected structures contained co-crystallized inhibitors, including clinically used DPP-4 inhibitors such as sitagliptin, vildagliptin, linagliptin, alogliptin, saxagliptin, teneligliptin, gosogliptin, and omarigliptin, as well as several synthetic inhibitors deposited in the PDB. The presence of a bound ligand in the crystal structure facilitates identification of the active site and enables a more reliable definition of the docking grid. In contrast, apo structures, such as 1J2E, 1NU6, and 5YP1, were used less frequently. Although apo structures may still be suitable for docking simulations, the absence of a bound ligand may complicate accurate identification of the binding pocket and introduce additional uncertainty in docking results [9].

Another notable observation is that most of the employed crystal structures exhibited relatively high resolution, typically ranging from approximately 1.6 Å to 2.6 Å, indicating that the majority of docking studies were performed using structurally reliable protein models. High-resolution structures are generally preferred for docking simulations because they provide more accurate atomic coordinates within the binding site.

Interestingly, in addition to commonly used and well-characterized structures, several studies employed less frequently used or unique PDB entries, which appeared only in single publications. In some cases, the rationale for selecting these particular structures was not clearly discussed in the corresponding articles. Such variability in the choice of protein models may contribute to methodological heterogeneity and may influence the comparability of docking results across different studies.

**Figure 6:**
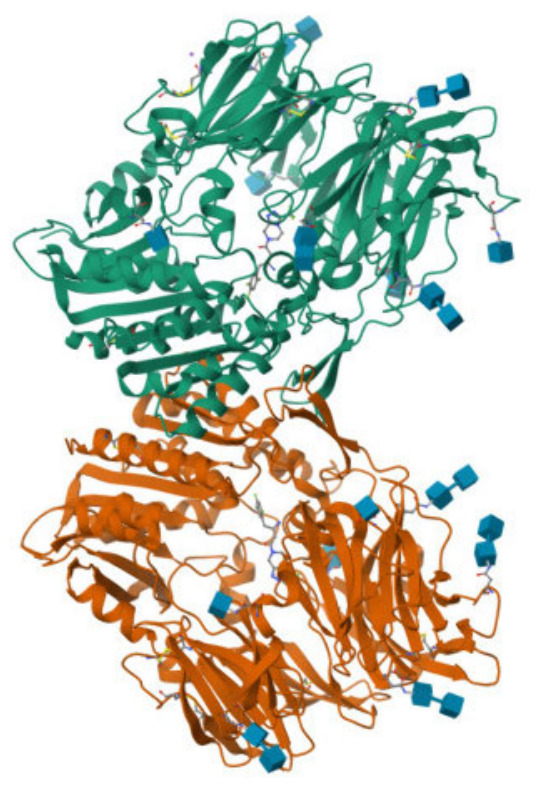
Crystal structure of dipeptidyl peptidase-4 (DPP-4) (PDB ID: 1X70) in complex with sitagliptin, a clinically approved DPP-4 inhibitor. This structure is widely used in molecular docking studies of natural compounds, as the presence of a co-crystallized ligand enables accurate definition and validation of the active site for computational analyses. Sourced from https://www.rcsb.org/.

The selection of an appropriate protein structure is therefore a critical step in molecular docking studies. Ideally, the chosen model should possess high crystallographic resolution and contain a well-defined binding pocket, preferably characterized by a co-crystallized ligand (Figure 6). Since the quality of the protein model directly affects docking outcomes, careful evaluation of available PDB entries prior to docking simulations is essential. Consequently, the process of selecting a suitable structural template should be performed thoughtfully and may require analysis of several available crystal structures.

## 5. Critical Assessment and Practical Guidelines for DPP-4 Docking Studies

### 5.1. Critical Limitations in Current Docking Approaches

Despite the widespread application of molecular docking in the identification of potential DPP-4 inhibitors, several important limitations can be identified across the reviewed studies.

First, a major issue is the lack of standardization in docking protocols. Considerable variability exists in protein structure selection, ligand preparation, grid definition, and scoring functions, which significantly affects the reproducibility and comparability of results. In many cases, the rationale behind the selection of a specific protein model or docking parameters is not clearly justified, further limiting the interpretability of the findings.

Second, a substantial proportion of studies rely exclusively on docking scores as indicators of inhibitory potential. However, docking scoring functions provide only approximate estimates of binding affinity and are known to produce false positives. In particular, hydrophobic and bulky natural compounds may artificially achieve favorable docking scores without demonstrating actual biological activity.

Third, insufficient validation represents a critical limitation. Only a limited number of studies perform redocking procedures, molecular dynamics simulations, or experimental verification of predicted interactions. As a result, many reported binding modes remain hypothetical and lack confirmation under dynamic or biological conditions. Another important challenge is related to the structural complexity of natural compounds. High conformational flexibility, multiple ionization states, and the presence of glycosylated moieties complicate accurate modeling and may lead to unrealistic docking poses if not properly addressed.

Finally, the static nature of molecular docking does not account for protein flexibility, solvent effects, or long-range interactions, all of which play a crucial role in ligand binding. This limitation is particularly relevant for DPP-4, which contains a relatively large and complex binding cavity with multiple subsites. Taken together, these limitations highlight that molecular docking should be considered a preliminary screening tool rather than a definitive method for predicting DPP-4 inhibitory activity. Integration with molecular dynamics simulations and experimental validation is essential to improve the reliability and translational value of computational predictions.

### 5.2. Practical Guidelines for Reliable Computational Studies

Based on the analysis of the reviewed literature, several key recommendations can be proposed to improve the quality, reproducibility, and translational relevance of molecular docking studies targeting DPP-4.

First, careful selection of the protein structure is essential. Whenever possible, high-resolution crystal structures co-crystallized with a ligand should be used, as they enable accurate identification of the active site and facilitate validation through redocking procedures. Structures such as 1X70 or 6B1E represent well-characterized and frequently used models in DPP-4 studies.

Second, validation of the docking protocol should be considered a mandatory step. Redocking of the co-crystallized ligand and evaluation of the root-mean-square deviation (RMSD) between experimental and predicted poses provide a basic measure of protocol reliability. Ideally, RMSD values below 2.0 Å should be achieved [9].

Third, ligand preparation requires particular attention, especially in the case of natural compounds. Proper consideration of protonation states, tautomeric forms, and conformational flexibility is necessary to avoid unrealistic docking results. For glycosylated or highly flexible molecules, additional conformational sampling may be required.

Another critical aspect is the definition of the docking grid. The grid box should be centered on the co-crystallized ligand or key catalytic residues and sufficiently large to cover all relevant subsites of the DPP-4 active site, including S1, S2, and S2 extensive regions. Improper grid definition may lead to incorrect binding modes.

Furthermore, docking results should not be interpreted solely on the basis of scoring functions. Detailed analysis of ligand–protein interactions, including hydrogen bonding, hydrophobic contacts, and π–π interactions with key residues such as Glu205, Glu206, Ser630, and Tyr662, is essential for meaningful interpretation.

Importantly, integration with complementary computational methods is strongly recommended. Molecular dynamics simulations can provide insight into the stability of the ligand–protein complex and account for protein flexibility and solvent effects, which are not captured in static docking models.

Finally, experimental validation should be considered an essential component of any docking-based study. Enzymatic inhibition assays, cell-based studies, or in vivo models are necessary to confirm the biological relevance of predicted interactions.

Overall, the implementation of standardized and well-validated computational workflows, combined with experimental verification, is crucial to enhance the reliability of molecular docking studies and support the development of effective natural DPP-4 inhibitors.

An additional observation emerging from the analyzed literature is the clear predominance of AutoDock Vina as the docking platform of choice. Its popularity is likely related to its open accessibility, ease of use, and relatively favorable balance between computational efficiency and prediction accuracy. However, the widespread use of a single software platform may also introduce systematic biases, as different scoring functions and search algorithms implemented in alternative programs may lead to distinct binding poses and affinity estimates. Therefore, comparison of docking scores obtained using different software should be performed with caution.

Another important source of variability is the choice of the protein structure used for docking. The present analysis demonstrated that ligand-bound crystal structures, particularly 1X70 and 6B1E, were employed considerably more frequently than apo structures. The presence of a co-crystallized ligand facilitates identification of the binding site and enables validation through redocking procedures, thereby increasing the reliability of docking calculations. In contrast, apo structures may introduce additional uncertainty associated with active-site definition and conformational variability.

Taken together, these observations emphasize that methodological heterogeneity remains one of the major challenges in the field. Differences in protein preparation, docking software, scoring functions, and validation strategies limit direct comparison of results reported by independent studies. Consequently, improved standardization of computational protocols would substantially enhance the reproducibility and translational value of molecular docking studies investigating natural DPP-4 inhibitors.

## 6. Natural Compounds Investigated as Potential DPP-4 Inhibitors

The studies reviewed in this section are organized according to major phytochemical classes, including flavonoids, phenolic compounds (with coumarins discussed separately), terpenoids and their subgroups (with saponins and sterols presented as distinct categories), as well as alkaloids and peptides.

The corresponding tables provide a structured overview of published studies in which molecular docking was applied to investigate these compounds as potential DPP-4 inhibitors. Given the substantial structural diversity both within and between these groups, their binding behavior toward DPP-4 cannot be directly compared in a uniform manner. Therefore, the following subsections summarize the main trends observed within each class, with particular emphasis on recurring interaction patterns and the extent of experimental validation supporting the computational findings.

### 6.1. Alkaloids

Alkaloids constitute a structurally diverse group of phytochemicals with increasing relevance in antidiabetic research, particularly in the context of DPP-4 inhibition (Table 3). Among them, isoquinoline alkaloids such as berberine and its derivatives appear to be the most consistently investigated, combining computational docking data with experimental enzyme inhibition studies.

For example, berberine has been reported in extracts of *Cardiospermum halicacabum* L. (Sapindaceae) based on HPLC and MS/MS analysis and was subsequently included in docking studies, which indicated potential DPP-4 inhibitory activity [16]. However, identification based solely on these techniques, without additional structural confirmation (e.g., NMR), may not be fully conclusive, introducing some uncertainty regarding the actual presence of this alkaloid in the plant material.

In contrast, protoberberine alkaloids are well-established constituents of *Coptis chinensis* Franch. (Ranunculaceae) and have been identified through integrated virtual screening and bioactivity evaluation approaches [24]. Overall, these findings support the potential of this structural class as a promising scaffold for natural DPP-4 inhibitor development.

The collected docking studies summarized in Table S2 further indicate that many alkaloids are predicted to bind within the catalytic region of DPP-4. Several reports describe interactions involving residues located in or near the active site, including Glu205, Glu206, Ser630, and Tyr662, although the reporting of specific binding interactions varies considerably among studies [23,24]. This variability reflects differences in docking protocols, selected protein structures, and computational platforms, which complicates direct comparison of binding affinities across publications.

Beyond protoberberine alkaloids, other structural classes have also been explored. Alkaloids such as withasomnine from *Withania coagulans* (Stocks) Dunal (Solanaceae) have been investigated using an integrated approach combining LC–MS-based phytochemical profiling, molecular docking and molecular dynamics simulations, in vitro DPP-4 inhibition assays, and in vivo evaluation in diabetic animal models. In vitro analysis of the fruit extract revealed a concentration-dependent inhibition of DPP-4 activity, reaching a maximum inhibition of 68.4% at a concentration of 60 mg/mL. For comparison, the reference inhibitor sitagliptin achieved a maximal inhibition of 90.1% under similar conditions. These findings were supported by stable ligand–protein interactions observed in silico, as well as improvements in glucose homeostasis and pancreatic histology in vivo [23]. Similarly, glycosin, an alkaloid reported from *Rhizophora apiculata* Blume (Rhizophoraceae), demonstrated antidiabetic activity in diabetic animal models supported by docking analyses, although further confirmation of the precise mechanism of action remains necessary [15].

Overall, integration of docking analyses with experimental findings indicates that alkaloids represent a promising but still incompletely characterized group of natural DPP-4 inhibitors. The dominance of protoberberine alkaloids in both computational and experimental studies suggests a particularly favorable structural framework for DPP-4 interaction [16,24]. However, inconsistencies in experimental validation, uncertainties regarding botanical sources in some reports, and methodological heterogeneity highlight the need for more standardized and integrated phytochemical, biochemical, and computational investigations [17,22].

### 6.2. Coumarins

Coumarins constitute a structurally diverse group of benzopyrone-derived phytochemicals that have attracted growing interest in antidiabetic research, particularly in relation to dipeptidyl peptidase-4 inhibition. Both simple coumarins and their glycosylated derivatives (e.g., cichoriin) have been investigated using molecular docking approaches, with several studies combining computational predictions with experimental enzyme inhibition assays. For instance, dicoumarol identified in *Schleichera oleosa* leaf extracts demonstrated measurable interactions with the DPP-4 active site in docking studies, with a binding affinity of approximately −7.8 kcal/mol and involvement of key residues such as Glu205, Glu206, Tyr662, and Ser630. In vitro experiments further indicated moderate inhibitory activity of the extracts (IC_50_ = 83–232 µg/mL), suggesting a potential contribution of coumarin-type compounds to the observed effects [19,28,29,30,31].

**Figure 7:**
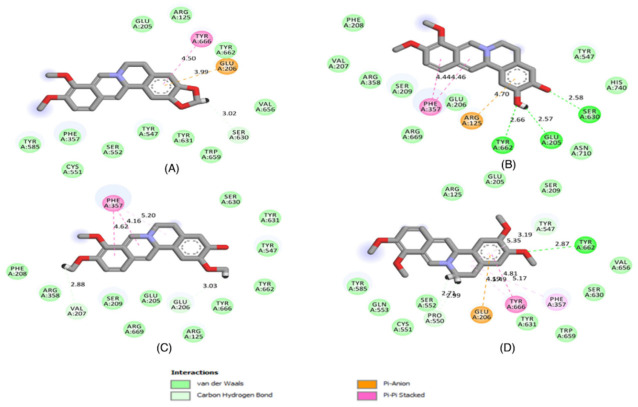
Binding interaction profile for DPP-IV and berberine compounds (**A**)-Berberine, (**B**)-Dimethylene berberin, (**C**)-Jatrorrhizine and (**D**)-New-isoform Berberine) obtained after molecular docking. Adapted with permission from [16].

Available docking studies generally indicate that coumarins are capable of occupying the catalytic region of DPP-4, frequently forming hydrogen bonds and hydrophobic interactions with residues associated with the S1 and S2 pockets of the enzyme. However, reported interaction profiles vary considerably between studies, reflecting differences in docking protocols, receptor preparation strategies, scoring functions, and computational platforms such as AutoDock, AutoDock Vina, or Molecular Virtual Docker [19,29,30]. This methodological heterogeneity complicates direct comparison of predicted binding affinities across publications. Moreover, compared with other classes of natural compounds, relatively few studies have specifically focused on the molecular docking of coumarins toward DPP-4. Consequently, the available evidence remains fragmented, and this structural class is still comparatively underexplored as a potential source of natural DPP-4 inhibitors (Figure 7, Table 4).

Beyond simple coumarins, glycosylated derivatives and dimeric structures such as dicoumarol have also been examined using combined computational and experimental approaches. Nevertheless, molecular dynamics simulations and consistent biochemical validation remain relatively limited, restricting firm conclusions regarding their inhibitory potency and pharmacological relevance as DPP-4 inhibitors [28,31].

Overall, current evidence suggests that coumarins may represent a promising yet still insufficiently characterized scaffold for natural DPP-4 inhibitor development. Although docking studies consistently indicate potential interactions within the catalytic site of the enzyme, further standardized computational workflows combined with systematic biochemical validation are required to clarify their therapeutic potential.

### 6.3. Flavonoids

Flavonoids are among the most abundant classes of plant secondary metabolites and occur widely in fruits, vegetables, and medicinal plants. Owing to their broad spectrum of biological activities, including antioxidant and antidiabetic effects, they have attracted considerable attention as potential inhibitors of dipeptidyl peptidase-4.

Flavonoids represent one of the most extensively investigated classes of natural compounds evaluated as potential DPP-4 inhibitors. Numerous docking studies summarized in Table 5 have explored a broad spectrum of flavonoid structures originating from various plant sources, reflecting both the chemical diversity of this group and its well-recognized biological activity. The analyzed studies include representatives of several flavonoid subclasses, such as flavonols (e.g., quercetin, kaempferol, isorhamnetin), flavones (apigenin, luteolin, cirsiliol), flavanones (hesperetin, naringenin), isoflavones (genistein, daidzein), flavan-3-ols (catechins), and anthocyanins and their glycoside derivatives.

In many studies, multiple flavonoids were evaluated within a single docking investigation, often derived from the same plant source, highlighting the interest in comparing structurally related compounds within this class. For example, Rath et al. [43] investigated several citrus-derived flavonoids such as naringin, hesperidin, naringenin, and quercetin using molecular docking approaches against diabetes-related targets including DPP-4.

Additionally, some studies explore structural modifications of natural flavonoid scaffolds; for instance, Kamboj et al. [60] designed and evaluated *O-methyl-substituted* quercetin analogs to assess how structural changes influence binding interactions within the DPP-4 active site.

Despite their structural diversity, several common interaction patterns with the DPP-4 catalytic site can be observed. Docking analyses frequently report hydrogen bonding and π–π interactions with residues located in the S1 and S2 pockets of the enzyme, particularly Glu205, Glu206, Ser630, Tyr547, Arg358, and Tyr662, which are known to play an important role in ligand recognition within the DPP-4 active site (Table S2).

**Figure 8:**
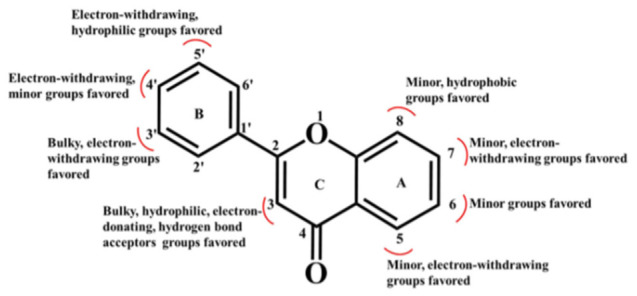
Structure−activity relationship of 30 flavonoids as dipeptidyl peptidase-IV inhibitors. Adapted with permission from [38].

Furthermore, compared with many other classes of natural compounds, a relatively large proportion of flavonoid docking studies include experimental validation, most commonly through DPP-4 inhibition assays performed in vitro, and in some cases supported by in vivo models. For example, Bhushan et al. [47] combined docking analysis with in vitro assays and in vivo studies in streptozotocin-induced diabetic mice to evaluate phytochemicals targeting DPP-IV. Their results demonstrated significant improvements in glycemic control, including a reduction in blood glucose levels by up to approximately 60% and favorable effects on lipid parameters. Docking analysis identified several flavonoid-type compounds, such as gallocatechin and astragalin, as potential DPP-4 inhibitors, exhibiting binding affinities in the range of −7 to −8.5 kcal/mol and interactions with key active-site residues, including Glu205 and Glu206. These findings support the potential contribution of flavonoid compounds to the observed antidiabetic effects, although additional mechanisms may also play a role (Figure 8).

In addition, some studies further strengthen docking predictions through molecular dynamics simulations, which allow assessment of the stability of ligand-protein complexes over time. An example of such an approach is provided by Ojo et al. [50], who performed molecular dynamics simulations following docking to evaluate the stability of ligand–protein interactions and the persistence of binding poses of bioactive plant compounds with diabetes-related targets, including DPP-4. Their results indicated that the analyzed complexes remained stable throughout the simulation, with low structural deviations and persistent hydrogen bond interactions, supporting the reliability of the predicted binding modes.

However, it should be noted that the available studies employ a wide range of docking protocols, including different docking software, scoring functions, receptor preparation strategies, and crystal structures of DPP-4 obtained from the Protein Data Bank. This methodological variability makes direct comparison of docking scores and predicted binding affinities between studies difficult. In addition, some flavonoids investigated in docking analyses are glycosylated derivatives characterized by relatively high molecular weight and polarity, which may limit their bioavailability despite favorable predicted binding interactions with the enzyme.

Overall, the reviewed studies indicate that flavonoids constitute one of the most promising and extensively investigated structural scaffolds among natural products evaluated as potential DPP-4 inhibitors.

### 6.4. Phenolics (Non-Flavonoid)

**Figure 9:**
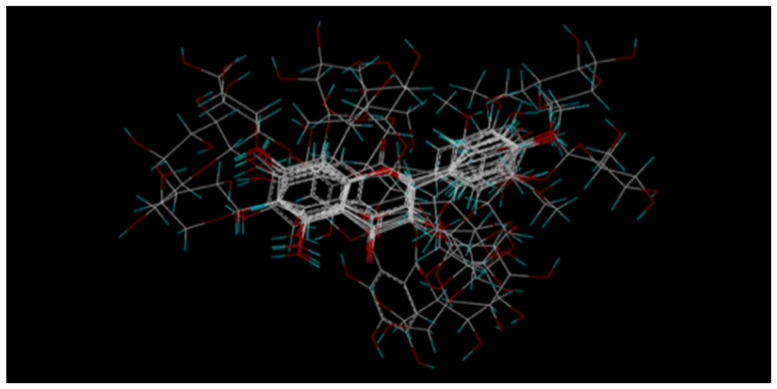
Molecular alignment of flavonoid compounds as dipeptidyl peptidase-IV inhibitor. Adapted with permission from [38].

Another group of phenolic compounds highlighted in this review includes phenolics that do not belong to the flavonoid class. For the purpose of this review, these compounds were grouped together as non-flavonoid phenolics, a category that encompasses a structurally diverse set of plant-derived molecules. This group includes simple phenolic acids (e.g., gallic acid, caffeic acid, ferulic acid, and *p*-coumaric acid), stilbenes such as resveratrol and piceatannol, as well as other phenolic derivatives including xanthones, lignans, curcuminoids, and glycosylated phenolics such as oleuropein and mangiferin. Many of these compounds are widely distributed in edible plants, fruits, and medicinal herbs and have been investigated for their potential antidiabetic properties, including the inhibition of DPP-4 (Figure 9).

Numerous docking studies summarized in Table 6 have evaluated phenolic compounds originating from a broad range of plant sources, including fruits, medicinal plants, and traditional herbal medicines. In several investigations, multiple phenolic compounds were screened simultaneously, particularly simple phenolic acids and their derivatives, in order to compare their binding potential toward the DPP-4 catalytic site. For example, Fan et al. [34] evaluated resveratrol together with several structurally related phenolic acids, including gallic and caffeic acids, to compare their inhibitory activity against DPP-4. Similarly, other studies have screened panels of phenolic compounds such as ferulic, sinapic, vanillic, and protocatechuic acids. Such comparative approaches enable the assessment of structure–activity relationships within closely related phenolic scaffolds by linking differences in chemical structure to variations in inhibitory activity.

Despite their structural diversity, many phenolic compounds were predicted to interact with key residues within the DPP-4 active site. Docking analyses frequently report hydrogen bonding and hydrophobic interactions involving residues located in the S1 and S2 pockets of the enzyme, particularly Glu205, Glu206, Ser630, Tyr547, Arg358, Tyr662, and Arg669. Several studies also reported π–π stacking interactions with aromatic residues such as Phe357 and Tyr547, which contribute to stabilization of ligand binding within the catalytic pocket (Table S2).

Compared with several other classes of natural compounds, phenolic compounds are relatively often evaluated in studies that combine molecular docking with experimental validation. In vitro DPP-4 inhibition assays are commonly used to confirm computational predictions, and some studies additionally include cellular or in vivo models of diabetes. For example, Sheela et al. [69] combined docking with gene expression analysis in HCT-15 cells and in vivo diabetic rat models, whereas Nuankaew et al. [72] investigated phenolic compounds using both enzyme inhibition assays and a zebrafish insulin-resistance model.

An interesting example of combining computational and experimental approaches is provided by Huang et al. [78], who evaluated several phenolic compounds including curcumin, resveratrol, syringic acid, ethyl gallate, and eugenol. In addition to molecular docking analysis, the authors performed DPP-4 inhibition assays and investigated the biological effects of selected compounds in cellular models, including C2C12, AR42J, and Caco-2 cells. Importantly, curcumin and resveratrol demonstrated direct inhibitory activity against DPP-4 in enzymatic assays, with curcumin achieving approximately 40% inhibition at 100 µM. In cellular models, curcumin further reduced DPP-4 activity in Caco-2 cells and modulated ERK phosphorylation in C2C12 cells. Moreover, in vivo studies revealed improved glucose tolerance and reduced HbA1c levels in diabetic mice following curcumin treatment. These findings suggest that DPP-4 inhibition may contribute to the observed biological effects, although additional mechanisms cannot be excluded [78].

Nevertheless, molecular dynamics simulations are still relatively rarely employed in studies focusing on phenolic compounds, although several recent investigations have begun to incorporate this approach to assess the stability of predicted ligand-protein complexes.

It should be noted that the category of phenolic compounds discussed in this section represents a structurally diverse group of compounds that were collectively analyzed within a single category for the purposes of this review. These compounds differ substantially in terms of their chemical structures, physicochemical properties, and binding capabilities, which limits the direct comparability of their docking results. Consequently, differences in reported binding affinities and interaction patterns should be interpreted with caution.

Despite this structural diversity and associated methodological limitations, the analyzed studies consistently indicate that phenolic compounds constitute a promising source of potential DPP-4 inhibitors, warranting further investigation and optimization. Notably, the most consistent and comprehensive evidence has been reported for compounds such as curcumin and resveratrol, which have been evaluated across multiple experimental levels, including enzymatic assays, cellular models, and in vivo studies, thereby supporting their potential biological relevance [40,78,83].

### 6.5. Saponins

Saponins are structurally complex amphiphilic glycosides typically composed of a triterpenoid or steroid aglycone linked to one or more sugar chains. Saponins have only recently begun to appear in studies investigating natural DPP-4 inhibitors, and the available literature remains noticeably limited compared with other phytochemical classes. Most reports focus on steroidal or triterpenoid saponins from medicinal plants such as *Trillium govanianum* Wall. ex D.Don (Melanthiaceae), *Gymnema sylvestre* (Retz.) R.Br. ex Sm. (Apocynaceae), or *Allium sativum* L. (Amaryllidaceae), where molecular docking was used primarily as a supportive tool alongside broader antidiabetic investigations (Table 7) [86,87,88].

In several cases, docking predictions were complemented by enzymatic DPP-4 inhibition assays or additional biological studies, suggesting that selected saponins may interact with regions close to the catalytic site of the enzyme. However, these experimental confirmations are not consistently reported across studies, and molecular dynamics simulations are only occasionally included, which limits the strength of mechanistic interpretation [22,89].

Another characteristic feature of this group is the considerable structural complexity of saponins, particularly their glycosylated moieties, which can influence docking outcomes and binding stability. Differences in docking software, scoring approaches, and protein preparation protocols further complicate cross-study comparisons, making it difficult to identify clear structure-activity trends.

Taken together, the currently available data suggest that saponins may represent an interesting but still insufficiently explored source of natural DPP-4 inhibitors. Importantly, while certain saponins exhibit antidiabetic effects, their activity toward DPP-4 appears to be moderate or insufficiently characterized [86]. The relatively small number of dedicated docking studies and the uneven level of experimental validation further indicate that this area remains at an early stage of investigation.

### 6.6. Sterols

Sterols are lipophilic tetracyclic triterpenoid-derived compounds characterized by a rigid steroid nucleus and variable aliphatic side chains. These structural features determine their strong hydrophobicity and relatively limited polarity, which may influence their interaction with protein targets such as DPP-4 (Table 8).

**Figure 10:**
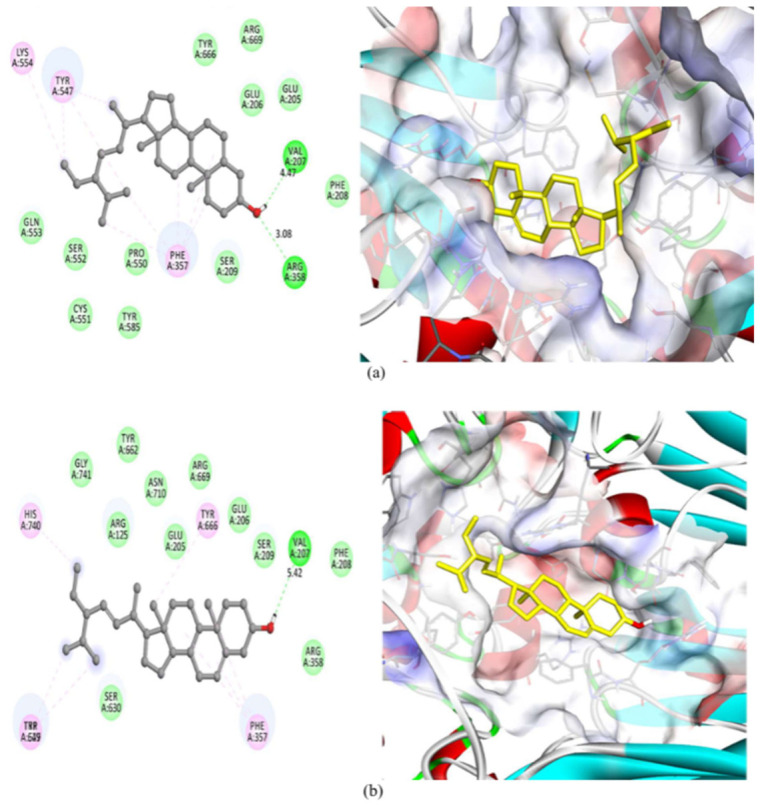
Molecular Docking of (**a**) β-sitosterol and (**b**) Stigmasterol into the Binding Site of Human DPP-IV. Adapted with permission from [91].

Across the available studies, molecular docking investigations most frequently focused on β-sitosterol and stigmasterol, which appear repeatedly in screenings of plant extracts [35,36,91,93]. Other sterols, including campesterol, α-spinasterol, and lanosterol, were examined less often and typically within broader phytochemical profiling rather than targeted inhibitor discovery [22,90,94]. In several studies, docking predictions were accompanied by enzymatic DPP-4 inhibition assays or antidiabetic biological evaluation, particularly for sterols isolated from *Urena lobata* L. (Malvaceae), *Pueraria tuberosa* (Roxb. ex Willd.) DC. (Fabaceae), or mixed plant extracts [35,36,93]. However, purely in silico studies without biochemical validation also remain common (Figure 10) [91,94].

A more comprehensive workflow was reported only in the study by Tangka et al. [90], where sterols from *Abelmoschus manihot* (L.) Medik. (Malvaceae) were evaluated using docking, molecular dynamics simulations, and enzymatic DPP-4 inhibition assays, providing a more coherent computational-experimental framework. However, the extract exhibited relatively weak inhibitory activity (IC_50_ = 860 µg/mL) compared to the reference inhibitor sitagliptin (IC_50_ = 9.7 µg/mL), and the contribution of individual compounds to the observed activity was not experimentally confirmed.

Molecular dynamics simulations were also reported in selected sterol-focused docking studies, performed using platforms such as YASARA and GROMACS, although these analyses remain relatively infrequent and methodologically heterogeneous across studies [22,90,92]. Such integrated approaches are otherwise relatively rare in this phytochemical group.

From a biological perspective, the pronounced lipophilicity and structural rigidity of sterols may favor hydrophobic accommodation within the DPP-4 binding pocket. However, these same features can also lead to overestimation of inhibitory potential in docking simulations, as hydrophobic fit does not necessarily translate into strong or specific enzymatic inhibition [90]. Consequently, although phytosterols occasionally demonstrate moderate computational affinity toward DPP-4, their role as standalone inhibitors remains uncertain due to the limited and inconsistent experimental validation. Overall, sterols appear to represent a supplementary and still relatively underexplored class of natural DPP-4 inhibitors, requiring more systematic experimental and mechanistic validation.

### 6.7. Terpenoids

Terpenoids constitute a broad and structurally diverse class of natural compounds derived from isoprene units, encompassing multiple subclasses such as monoterpenoids, sesquiterpenoids, diterpenoids, and triterpenoids, as well as structurally related derivatives. The compounds included in Table 9 represent several of these subclasses, highlighting the wide chemical diversity within this group. These molecules differ substantially in terms of size, functional groups, polarity, and three-dimensional structure, which directly influences their binding behavior toward protein targets.

Due to this structural diversity, terpenoid compounds have been investigated as potential DPP-4 inhibitors using a range of complementary approaches. Several studies have combined molecular docking analyses with molecular dynamics simulations and, in selected cases, in vitro or in vivo experiments, enabling a more comprehensive evaluation of their potential inhibitory activity.

For instance, phytoconstituents identified in *Withania coagulans* demonstrated significant DPP-4 inhibition both in vitro and in diabetic animal models, accompanied by improvements in glucose homeostasis parameters and restoration of pancreatic tissue morphology. Molecular docking and ADMET analyses further supported the favorable binding and drug-like properties of the identified compounds [113].

Similarly, terpenoid-rich extracts from *Amberboa ramosa* (Roxb.) Jafri (Asteraceae) exhibited inhibitory activity against DPP-4, with enzymatic assays and kinetic studies indicating a mixed mode of inhibition. Molecular docking identified valeranone as a key compound interacting within the catalytic pocket, while molecular dynamics simulations confirmed the stability of the protein-ligand complex [96].

An interesting extension of this group includes cannabinoid compounds, which, although not classified as classical terpenoids, are structurally derived from terpenoid precursors and were therefore included in this section. Experimental studies revealed that compounds such as cannabidiol (CBD), cannabigerol (CBG), and cannabinol (CBN) exhibit dose-dependent inhibition of DPP-4, with reported IC_50_ values in the range of approximately 4.0–6.9 μg/mL, depending on the compound. Molecular docking and molecular dynamics simulations further confirmed stable interactions with both active and allosteric sites of the enzyme [110].

Despite these promising findings, molecular dynamics simulations are still not consistently applied across studies on terpenoids, and experimental validation remains uneven. Given the substantial structural heterogeneity within this group, comparisons of docking scores and binding affinities across individual compounds remain inherently limited.

Nevertheless, the available evidence suggests that terpenoids constitute a promising and mechanistically diverse class of natural DPP-4 inhibitors, particularly when supported by integrated computational and experimental approaches.

### 6.8. Peptides

In addition to small-molecule natural compounds, a growing number of studies have investigated peptides as potential DPP-4 inhibitors using molecular docking approaches. This trend reflects the increasing interest in food-derived and bioactive peptides as functional agents in metabolic disorders, particularly in the context of type 2 diabetes.

A substantial proportion of these studies focus on short peptide sequences derived from dietary proteins, including legumes (e.g., *Glycine max* (L.) Merr., *Phaseolus vulgaris* L.) [121,122], cereals, and other plant-based sources. Molecular docking is frequently employed as an initial screening tool to identify peptide sequences with potential affinity toward the DPP-4 active site prior to experimental validation.

Notably, compared with small-molecule studies, peptide-based investigations often rely on simpler and more standardized docking workflows, most commonly using AutoDock or AutoDock Vina, and are frequently complemented by in vitro DPP-4 inhibition assays. However, molecular dynamics simulations are still rarely applied in peptide docking studies, similarly to observations made for small-molecule ligands.

Despite the increasing number of publications in this area, peptide docking studies are often focused on screening large sets of sequences rather than detailed structure-activity relationship analysis. As a result, the available data remain highly heterogeneous and difficult to compare across studies.

Due to the large number and diversity of investigated peptide sequences, detailed information on individual studies has been provided in the Supplementary Material (Table S1) [121,122,123,124,125,126,127,128,129,130,131,132,133,134,135,136,137,138,139,140,141,142,143,144,145,146,147].

### 6.9. Overall Trends in Experimental Validation

Among the 178 studies analyzed, molecular dynamics simulations were performed only in a minority of investigations, confirming that most reports relied exclusively on docking calculations. Experimental validation was heterogeneous and ranged from isolated DPP-4 inhibition assays to cell-based studies and in vivo models. However, DPP-4 inhibition assays were considerably more common than cellular or animal experiments, whereas several studies reported broader antidiabetic effects without demonstrating direct inhibition of DPP-4. Therefore, the biological significance of favorable docking scores should be interpreted with caution, since antidiabetic activity may arise from mechanisms unrelated to DPP-4 inhibition (Table 10).

## 7. Structure–Activity Relationships of Natural DPP-4 Inhibitors

Analysis of the interaction patterns summarized in the reviewed studies indicates that natural compounds share several key binding features with clinically used DPP-4 inhibitors. In particular, hydrogen bonding with the catalytic residues Glu205 and Glu206 appears to be a frequently observed and functionally important interaction motif across multiple classes of natural compounds, including flavonoids, phenolics, and alkaloids [34,37,38,39,41]. Notably, these residues are also central to the binding of marketed gliptins, which consistently form hydrogen bonds with Glu205 and Glu206, confirming their essential role in ligand anchoring within the active site [4]. In addition, frequent interactions with residues such as Arg358 and Arg669 suggest a potential contribution of electrostatic and hydrogen-bonding interactions to ligand recognition.

In addition to hydrogen bonding, π–π stacking interactions with aromatic residues such as Tyr547, Tyr662, and Phe357 are frequently observed, highlighting the importance of aromatic ring systems for stabilizing ligand binding within the S1 pocket [39,41,70]. This is in agreement with interaction profiles reported for synthetic DPP-4 inhibitors, where aromatic moieties contribute to hydrophobic and π-mediated interactions within the binding cavity [4]. Interactions with catalytic residues such as Ser630 are also reported, although less consistently, suggesting a more limited engagement of the catalytic machinery compared to synthetic inhibitors [19,73].

Despite these similarities, natural compounds generally exhibit less complex and less optimized interaction networks compared to gliptins. While synthetic inhibitors are designed to occupy multiple subsites within the DPP-4 active site and maximize interactions across S1, S2, and additional regions, natural compounds are most often associated with residues located within the S1 and S2 pockets, with only occasional extension toward the S2 extensive subsite [71]. This more limited and less coordinated binding pattern may contribute to their generally lower reported binding affinities and highlights the importance of structural optimization in the development of potent DPP-4 inhibitors.

A comprehensive overview of residue-level interactions for all analyzed compounds is provided in the Supplementary Material (Table S2), where detailed interaction profiles derived from individual docking studies are compiled.

## 8. Limitations and Future Perspectives of Docking Studies on Natural DPP-4 Inhibitors

Despite the growing number of molecular docking studies investigating natural compounds as potential DPP-4 inhibitors, several methodological limitations can be identified across the current literature, which may affect the reliability and interpretability of reported results. At the same time, it is important to recognize that the increasing application of docking approaches in studies on natural products reflects a broader trend in modern drug discovery, where computational methods are used to accelerate the identification of promising bioactive compounds and reduce the time and cost associated with experimental screening.

A critical factor influencing docking outcomes is the selection of an appropriate protein structure. The choice of PDB entry, including its resolution, conformational state, and the nature of the co-crystallized ligand, can significantly impact predicted binding modes. Structures containing well-characterized inhibitors in the active site are generally preferred, as they better reflect biologically relevant binding conformations. In this context, validation of docking protocols using multiple PDB structures may provide a more robust assessment of ligand binding and reduce structure-dependent bias.

Another major limitation is the inconsistent application of molecular dynamics simulations. While docking provides a rapid estimation of binding modes, it remains a static approach that does not account for protein flexibility or solvent effects. Therefore, MD simulations should be considered an essential step in validating docking results, particularly when proposing stable binding interactions or mechanistic interpretations.

Furthermore, substantial variability exists in docking methodologies across studies, including differences in software platforms, scoring functions, and protein preparation procedures. These methodological discrepancies can lead to inconsistencies in predicted binding affinities and interaction patterns, limiting the comparability of results between independent studies.

Importantly, experimental validation remains insufficient in many reports. Although docking can serve as a valuable tool for the initial screening of large numbers of compounds, it provides only a theoretical estimation of binding and does not confirm biological activity. Therefore, enzymatic assays, cellular studies, and in vivo models are essential to verify the inhibitory potential of identified compounds. In this regard, molecular docking should be regarded primarily as a hypothesis-generating and screening approach rather than a definitive method for confirming biological activity.

In the context of natural products, additional challenges should also be considered. Many plant-derived compounds, particularly polyphenols such as flavonoids and certain alkaloids, have demonstrated promising interactions with the DPP-4 active site in docking studies. Among these, the most consistently investigated and biologically supported compounds include protoberberine alkaloids such as berberine and its derivatives, as well as selected flavonoids, including quercetin and gallocatechin, and other phenolic compounds such as resveratrol and curcumin. However, their pharmacokinetic properties, including limited bioavailability, rapid metabolism, and extensive biotransformation after absorption, may significantly affect their actual biological activity. Consequently, strong docking scores do not necessarily translate into in vivo efficacy.

Despite the growing number of reports describing favorable docking interactions between natural compounds and DPP-4, successful translation into therapeutic agents requires consideration of additional factors beyond binding affinity. Many phytochemicals exhibit limited aqueous solubility, poor membrane permeability, low oral bioavailability, or extensive first-pass metabolism, which may significantly restrict their clinical utility. Therefore, favorable docking scores should not be interpreted as direct indicators of drug-likeness or therapeutic efficacy.

Future investigations should integrate molecular docking with complementary approaches addressing pharmacokinetic and biopharmaceutical properties, including ADME predictions, molecular dynamics simulations, and formulation strategies aimed at improving solubility and bioavailability. Such integrated approaches may enhance the translational potential of natural compounds identified as promising DPP-4 inhibitors.

**Figure 11:**
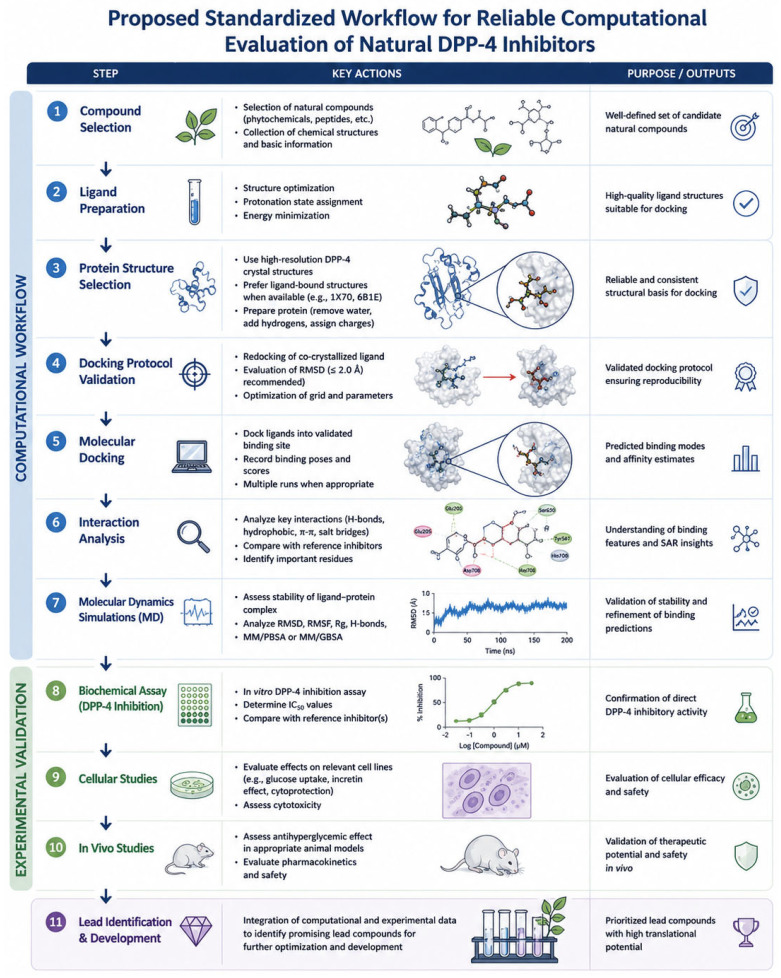
Proposed standardized workflow for future studies on natural DPP-4 inhibitors. Based on the trends and limitations identified in the analyzed literature, the workflow combines computational approaches, including protein structure selection, docking protocol validation, molecular docking, interaction analysis, and molecular dynamics simulations, with progressively higher levels of experimental verification. Such an integrated strategy may facilitate quantitative comparison of compounds and enhance the reliability and translational value of docking-based studies.

Based on the analysis of the reviewed literature, a standardized workflow (Figure 11) for the computational evaluation of natural DPP-4 inhibitors can be proposed. Such an approach should include the use of high-resolution ligand-bound crystal structures, validation of docking protocols through redocking procedures, detailed analysis of ligand–protein interactions, and, whenever possible, molecular dynamics simulations. Importantly, computational predictions should be complemented by experimental verification, including biochemical DPP-4 inhibition assays, cellular studies, and in vivo investigations. Adoption of more standardized workflows would facilitate comparison of results obtained by independent groups and improve the translational relevance of docking-based studies.

Taken together, these considerations highlight that, while molecular docking represents a powerful and increasingly utilized tool in the search for novel DPP-4 inhibitors from natural sources, its results should be interpreted critically and in conjunction with complementary computational and experimental approaches. When applied appropriately, docking can substantially support early-stage screening and guide further investigation, but it should not be used as a standalone method for drawing definitive conclusions.

## 9. Conclusions

This review summarizes key findings from 178 studies investigating molecular docking of natural compounds as potential inhibitors of dipeptidyl peptidase-4, providing a broad overview of current computational approaches in this field. The collected data clearly demonstrate a growing interest in the use of in silico methods for the identification of natural compounds with potential antidiabetic activity.

Across the analyzed studies, natural products emerge as a structurally diverse and promising source of DPP-4 inhibitors, with phenolic compounds and alkaloids representing the most consistently investigated and supported groups. At the same time, the literature reveals noticeable variability in computational methodologies, including differences in docking software, scoring functions, and protein structure selection.

Importantly, the findings of this review highlight the need for more standardized and rigorously validated computational workflows. In particular, the integration of molecular dynamics simulations and consistent experimental validation remains limited, despite their critical role in improving the reliability of docking predictions.

Overall, molecular docking represents a valuable and efficient tool for early-stage screening of natural compounds. However, its results should be interpreted with caution, especially considering the complexity of natural products, including issues related to bioavailability and metabolic stability. The future development of this field will depend on the combination of computational strategies with experimental approaches, enabling more reliable identification of biologically relevant DPP-4 inhibitors.

## Figures and Tables

**Table 1:** Docking tools employed in DPP-4 studies.

Software	Number of Studies
AutoDock	32
AutoDock Vina	56
CB Dock	3
CDOCKER	7
Dockingserver.com	2
Discovery Studio	4
FlexX	2
FRED	3
Glide	18
GOLD	3
Molecular Operating Environment (MOE)	6
Molegro Virtual Docker	10
PLANTS	2
SYBYL	5
Yasara	2
Other	23

**Table 2:** Protein structures of DPP-4 used in docking studies.

PDB ID	Resolution (Å)	Co-Crystallized Ligand	Number of Studies
1J2E	2.60	None (apo structure)	3
1NU6	2.60	None (apo structure)	3
1NU8	2.50	Diprotin A	3
1RWQ	2.20	PDB ligand ID: 5AP (synthetic DPP-4 inhibitor) DrugBank ID: DB02004	2
1WCY	2.20	Diprotin A	10
1X70	2.10	Sitagliptin	24
2BGR	2.00	HIV-1 Tat(1-9) derived peptide	1
2G63	2.00	Cyanopyrrolidine (C5-Pro-Pro) inhibitor 24b	4
2I03	2.40	Alkynyl cyanopyrrolidine inhibitor (ABT-279)	3
2ONC	2.55	Alogliptin	11
2P8S	2.20	Cyclohexalamine inhibitor	5
2QT9	2.10	PDB ligand ID: 524 (synthetic DPP-4 inhibitor) DrugBank ID: DB07135	1
2RGU	2.60	Linagliptin	1
2RIP	2.90	34Q-(3R,4R)-4-(pyrrolidin-1-ylcarbonyl)-1-(quinoxalin-2-ylcarbonyl)pyrrolidin-3-amine	4
3BJM	2.35	Saxagliptin	1
3F8S	2.43	Gosogliptin	5
3G0B	2.25	Alogliptin	7
3KWF	2.40	Carmegliptin	1
3VJK	2.49	Teneligliptin	2
3VJM	2.10	PDB ligand ID: W61 (synthetic DPP-4 inhibitor)	2
3W2T	2.36	Vildagliptin	3
3WQH	2.85	Anagliptin	4
4A5S	1.62	PDB ligand ID: N7F (synthetic DPP-4 inhibitor)	12
4FFV	2.40	11A19 Fab (antibody fragment)	1
4FFW	2.90	Sitagliptin + Fab fragment	3
4J3J	3.20	PDB ligand ID: D3C (synthetic DPP-4 inhibitor)	3
4LKO	2.43	PDB ligand ID: 1WH (synthetic DPP-4 inhibitor)	1
4N8D	1.65	PDB ligand ID: 2KS (synthetic DPP-4 inhibitor)	3
4N8E	2.30	PDB ligand ID: 2KV (synthetic DPP-4 inhibitor)	1
4PNZ	1.90	Omarigliptin	6
5J3J	2.75	PDB ligand ID: HL1 (synthetic DPP-4 inhibitor)	5
5KBY	2.24	Trelagliptin	1
5T4B	1.76	PDB ligand ID: 75N (synthetic DPP-4 inhibitor)	5
5T4E	1.77	PDB ligand ID: 75L (synthetic DPP-4 inhibitor)	1
5T4F	1.90	PDB ligand ID: 75M (synthetic DPP-4 inhibitor)	2
5Y7H	3.00	PDB ligand ID: 8O3 (synthetic DPP-4 inhibitor)	2
5Y7J	2.52	PDB ligand ID: 8OL (synthetic DPP-4 inhibitor)	1
5Y7K	2.51	PDB ligand ID: 8VU (synthetic DPP-4 inhibitor)	5
5YP1	2.47	None (apo structure)	1
5YP3	2.44	Ile-Pro from Pseudoxanthomonas mexicana	1
6B1E	1.77	Vildagliptin	14
6B1O	1.91	Vildagliptin Analog	2
Not specified			5
Homology model			1

**Table 3:** Alkaloids docked to DPP-4.

Compound	Reported Natural Source	PDB ID	Docking Software	MD *	Experimental Validation	Reference
Berberine	*Coptis chinensis*	2G63	FRED	−	DPP-4 Inhibition Assay	[13]
Berberine Australine Castanospermine	*Castanospermum australe*	-	GOLD	−	DPP-4 Inhibition Assay	[14]
Glycosin	*Rhizophora apiculata*	2RIP	AutoDock 4.0	−	In vivo (STZ/nicotinamide diabetic rat)	[15]
Berberine	*Cardiospermum halicacabum*	6B1E	AutoDock Vina	+	DPP-4 Inhibition Assay	[16]
Berberine Palmatine	*Fibraurea tinctoria*	6B1E	AutoDock 4.0	−	In vitro antioxidant activity test	[17]
Tetrandrine Limacusine	*Phaeanthus ophtalmicus*	2RIP	UCSF Chimera	−	DPP-4 Inhibition Assay	[18]
Colchicine	*Schleichera oleosa*	5T4B	AutoDockTools v.1.5.7	+	DPP-4 Inhibition Assay	[19]
Anonaine	*Annona squamosa*	4A5S	AutoDock software (v4.2.6)	−	-	[20]
Elaeocarpidine Elaeocarpine	*Elaeocarpus serratus*	4A5S	AutoDock software (v4.2.6)	−	-	[20]
Actinodaphnine Coreximine	*Litsea glutinosa*	3F8S	AutoDockVina version 1.1.2	−	-	[21]
Adunctin C	*Piper aduncum*	2ONC	AutoDock 4.2 and AutoDock Vina	+	-	[22]
Veramiline	*Eclipta prostata*	2ONC	AutoDock 4.2 and AutoDock Vina	+	-	[22]
Withasomnine	*Withania coagulans*	5Y7K	AutoDock	+	DPP-4 Inhibition Assay; in vivo STZ-nicotinamide T2DM rat model	[23]
Berberine Coptisine Jatrorrhizine	*Coptis chinensis*	5Y7H	Surflex-Dock (SYBYL)	+	DPP-4 Inhibition Assay	[24]
Campthotecin	*Garcinia atroviridis*	2RIP	AutoDock Vina	+	DPP-4 Inhibition Assay; in ovo assays; cytotoxicity testing	[25]
Corydine Lauroscholtzine	*Dalbergia sissoo*	1X70	AutoDock Vina	+	-	[26]
Solanocapsine	Combinations of extracts from *Siraitia grosvenorii*, *Dimocarpus longan* and *Orthosiphon stamineus*	6B1E	Glide XP	+	DPP-4 Inhibition Assay; in vitro (L6 cells); cytotoxicity	[27]

* MD: molecular dynamics simulation; ‘+’ indicates that MD simulation was performed; ‘−’ indicates that MD simulation was not performed.

**Table 4:** Coumarins docked to DPP-4.

Compound	Reported Natural Source	PDB ID	Docking Software	MD	Experimental Validation	Reference
Coumarin	Not reported	4J3J	Molegro Virtual Docker 5.0 2012	−	DPP-4 Inhibition Assay	[28]
Coumarin	*Cinnamomum burmannii* and *Caesalpinia sappan*	1X70	AutoDock 4.2.6	−	DPP-4 Inhibition Assay	[29]
Scopoletin (6-methoxy-7-hydroxy coumarin)	*Lunasia amara*	5Y7K	Pyrx software version 2.2.3	−	-	[30]
Dicoumarol	*Schleichera oleosa*	5T4B	AutoDockTools v.1.5.7	+	DPP-4 Inhibition Assay	[19]
Cichoriin (6-hydroxy-7-*O*-glucosylcoumarin)	*Fraxinus hupehensis*, *Calea fruticosa*	1J2E	AutoDock Vina (version 1.1.2)	−	DPP-4 Inhibition Assay; cytotoxicity; in vitro	[31]
Toddalolactone	*Toddalia asiatica*	5T4B	ArgusLab 4.0.1	−	-	[32]

‘+’ indicates that MD simulation was performed; ‘−’ indicates that MD simulation was not performed.

**Table 5:** Flavonoids docked to DPP-4.

Compound	Reported Natural Source	PDB ID	Docking Software	MD	Experimental Validation	Reference
Naringin (naringenin 7-*O*-neohesperidoside)	*Citrus* spp.	2ONC	Molegro Virtual Docker 4.1.0 trial version.	−	DPP-4 Inhibition Assay; in vitro; in vivo; ELISA	[33]
Apigenin Genistein Hesperetin Kaempferol Naringenin Quercetin	*Citrus* spp. and *berries* (*Vaccinium corymbosum*, *Rubus fruticosus*)	2I03	AutoDock 4.2	−	DPP-4 Inhibition Assay	[34]
Gossypetin Chrysoeriol	*Urena lobata*	-	www.dockingserver.com	−	DPP-4 Inhibition Assay	[35]
Daidzin Robinin (*O*-glycoside)	*Pueraria tuberosa*	4FFV	YASARA	−	DPP-4 Inhibition Assay; in vivo diabetic rat model	[36]
Kaempferol *O*-glycosides	*Lens culinaris*	1X70	Glide	−	DPP-4 Inhibition Assay	[37]
Quercetin	Not reported	4J3J	Molegro Virtual Docker 5.0 2012	−	DPP-4 Inhibition Assay	[28]
Isorhamnetin -*O*-glycosides Cyanidin-3-*O*-glucoside	Not reported	1X70	SYBYL (Surflex-Dock)	−	DPP-4 Inhibition Assay; Caco-2 expression study	[38]
Rutin	Not reported	2ONC	Glide	+	-	[39]
Taxifolin	Not reported	4A5S	Surflex-Doc SYBYL-X 1.3	+	DPP-4 Inhibition Assay	[40]
Rutin (quercetin-3-*O*-rutinoside) Quercetin-3-*O*--glucoside Naringin Kaempferol Chalcone	*Solanum elaeagnifolium*	2RIP	Glide	−	In Vitro Antidiabetic Activity	[41]
Luteolin Apigenin Pinocembrine	*Crescentia cujete*	1WCY	AutoDock Vina	+	-	[42]
Diosmetin Diosmin (diosmetin 7-*O*-rutinoside) Hesperidin Naringenin Naringin Nobiletin Quercetin Sudachitin Tangeretin	Not reported	5T4E	AutoDock 4.2	−	-	[43]
Gallocatechin (+)-Catechin (−)-Epicatechin Taxifolin Procyanidin B1	*Prunus persica*	1X70	AutoDock Vina	−	DPP-4 Inhibition Assay	[44]
Quercetin Kaempferol	Not reported	1J2E	AutoDock Vina 1.1.2 software	−	In vivo diabetic rat model, cell viability assay	[45]
Genistein Glycitin (glycitein 7-*O*-glucoside) Genistein 8-*C*-glucoside Aureusidin	*Chrozophora rottleri*	4A5S	AutoDock Vina	−	-	[46]
Gallocatechin Astragalin (kaempferol 3-*O*-glucoside) Quercetol C	*Phyllantus niruri*	4A5S	Glide	−	In vivo antidiabetic study (STZ-induced diabetic mice model)	[47]
Quercetin Kaempferol Isorhamnetin Fisetin Naringenin Apigenin Luteolin Myricetin Puerarin Biochanin A Rutin Anthocyanin Silymarin	Not specified	1X70	CB-Dock	−	-	[48]
Luteolin Apigenin Petunidin	*Syzygium cumini* *Ocium sanctum* *Psidium guajave*	5Y7H	Glide	+	-	[49]
Myricetin Rutin Quercetrin (Quercetin 3-*O*-rhamnoside)	Beta vulgaris Persea americana Syzygium aromaticum	6B1E	AutoDock Vina	+	DPP-4 Inhibition Assay; cytotoxicity	[50]
Chrysin	Not reported	6B1E	FlexX	−	DPP-4 Inhibition Assay	[51]
Rutin Naringin Hesperidin (hesperetin 7-*O*-rutinoside) Naringenin Eriocitrin (eriodictyol-7-*O*-rutinoside) Hesperetin Eriodictyol	Citrus bioflavonoids (commercial nutraceuticals)	2ONC	AutoDock Vina	−	DPP-4 Inhibition Assay	[52]
5,7-dihydroxy-6-4-dimethoxyflavanone Homoesperetin-*O*-7-rutinoside	*Chromolaena odorata*	6B1O	AutoDock Vina	−	In vivo GLP-1 modulation study (rats)	[53]
Catechin	*Withania somnifera*	1NU6	PatchDock	−	DPP-4 Inhibition Assay; STC-1 cell assay; fluorescence/CD spectroscopy	[54]
Galangin	Not specified	6B1E	FlexX tool	−	DPP-4 Inhibition Assay; L6 cell culture study	[55]
5,3′,4′-trihydroxy-6,7-dimethoxyflavone Quercetagetin-3,4′-dimethyl ether	*Melicope glabra*	1X70	AutoDock Vina 1.5.6	−	DPP-IV Inhibition Assay	[56]
Catechin Genistein Robustaflavone Kaempferol Quercetin Myricetin Apigenin	*Anacardium occidentale*	2ONC	AutoDock Vina	−	-	[57]
Hyperoside (quercetin-3-*O*-galactoside) Myricetin Narcissoside (Isorhamnetin-3-*O*-rutinoside) Cyanidin-3-*O*-glucoside Isoliquiritigenin	Not specified	1X70	AutoDock 4.2 software	−	DPP-IV Inhibition Assay	[58]
Hibiscetin	*Hibiscus cannabinus*	1RWQ	Glide	−	-	[59]
*O*-methyl quercetin analogs	Synthetic derivatives of quercetin	4J3J	Molegro Virtual Docker 6.0	−	-	[60]
Cirsiliol Cirsimarin (cirsimaritin-4′-*O*-glucoside) Cirsimaritin Pedalitin	*Ruellia tuberosa*	4A5S	Glide	−	-	[61]
Isorhamnetin	Not specified	4A5S	AutoDock Vina	+	-	[62]
Glycitein Pectolinarigenin Formononetin	*Peronema canescens*	3G0B	Molegro Virtual Docker 5.0	−	DPP-4 Inhibition Assay	[63]
Epigallocatechin-3-*O*-gallate Gallocatechin-3-*O*-gallate	Catechins from dietary plant sources	4LKO	Flare™ software	+	-	[64]
Salvigenin Daidzein	*Ocimum gratissimum* *Jatropha curcas*	4A5S	AutoDock Vina (PyRx)	+	-	[65]
Rutin Hesperidin	*Citrus aurantiifolia*	3G0B	Molegro Virtual Docker 5.0	−	-	[66]
Quercetin Kaempferol Luteolin Rutin	*Abelmoschus esculentus*	2ONC	AutoDock Vina	−	DPP-4 Inhibition Assay	[67]
Cyanidin glycosides Luteolin glycosides Isorhamnetin glycosides Quercetin and derivatives	*Brassica oleracea*	1NU6	AutoDock Vina 4.2	−	-	[68]

‘+’ indicates that MD simulation was performed; ‘−’ indicates that MD simulation was not performed.

**Table 6:** Phenolics (non-flavonoid) docked to DPP-4.

Compound	Reported Natural Source	PDB ID	Docking Software	MD	Experimental Validation	Reference
Caffeic acid Gallic acid Resveratrol	*Citrus* spp. and berries	2I03	AutoDock 4.2	−	DPP-4 Inhibition Assay	[34]
Caffeic acid Gallic acid	*Cocos nucifera*	3F8S	CDOCKER	−	Cell line study (HCT-15); in vivo diabetic rat model	[69]
Oleacein Oleocanthal Oleuropein	*Olea europaea*	5T4F	PLANTS	−	DPP-4 Inhibition Assay; Caco-2 expression study	[70]
4-hydroxybenzaldehyde Caffeic acid 4-*O*-glucoside Gallic acid *p*-Coumaric acid	*Bambusa arundinacea Oryza sativa*	2I03	AutoDock 4.2	−		[71]
Chrysophanol Dihydropiceatannol Emodin Piceatannol Resveratrol	*Senna siamea*	1J2E	AutoDock Vina 1.1.2	−	DPP-4 Inhibition Assay; zebrafish larvae insulin-resistance model	[72]
Oleuropein	Not reported	2ONC	Glide	+	-	[39]
Calebin A	*Curcuma longa*	3VJK	Glide	+	DPP-4 Inhibition Assay	[73]
Resveratrol	Not reported	4A5S	Surflex-Doc SYBYL-X 1.3	+	DPP-4 Inhibition Assay	[40]
Peperochromene A	*Peperomia pellucida*	4PNZ	AutoDock 4.2	−	-	[74]
Chlorogenic acid Cinnamic acid Ferulic acid Salicylic acid Sinapic acid	*Solanum elaeagnifolium*	2RIP	Glide	−	In Vitro Antidiabetic Activity	[41]
Cryptochlorogenic acid Ellagic acid Isoferulic acid Vanillic acid α-Hydrojuglone glucoside	*Chrozophora rottleri*	4A5S	AutoDock Vina	−	-	[46]
Brevifolin carboxylic acid Ellagic acid Gallic acid Methyl brevifolincarboxylate	*Phyllantus niruri*	4A5S	Glide	−	In vivo antidiabetic study (STZ-induced diabetic mice model)	[47]
Phenanthrene glycosides	*Elatostema tenuicaudatum*	4FFW	CDOCKER	−	HepG2 cell-based study	[75]
Mangiferin	*Mangifera indica*	2P8S	AutoDock Vina 1.1.2	+	DPP-4 Inhibition Assay	[76]
Sennoside A	*Cassia angustifolia*	2P8S	Glide	+	DPP-4 Inhibition Assay	[77]
Benzyl cinnamate Curcumin Ethyl gallate Eugenol Resveratrol Syringic acid	Not reported	2ONC	AutoDock	−	DPP-4 Inhibition Assay; Cell culture (C2C12, AR42J, Caco-2)	[78]
Gallic acid	*Trigonella foenum*	3F8S	AutoDock	−	DPP-4 Inhibition Assay; in vivo diabetic rat model (corticosteroid-induced T2DM)	[79]
α-mangostin γ-mangostin xanthone	*Garcinia mangostana*	3W2T	MOE	−	-	[80]
Caffeic acid Ferulic acid Gallic acid *p*-coumaric acid Protocatechuic acid Salicylic acid Sinapic acid Vanillic acid	*Anacardium occidentale*	2ONC	AutoDock Vina	−	-	[57]
Syringaldehyde	*Tetradium ruticarpum* *Blumea lanceolaria* *Microtropis japonica* *Coix lacryma-jobi,* *Phaius Mish-mensis* *Garcinia lini*	4N8D	GOLD	−	In vivo DIO diabetic mouse model (OGTT, ITT)	[81]
Matairesinol	Not specified	-	Mcule software	−	In vivo diabetic rat model	[82]
Curcumin	Not specified	2P8S	SYBYL-X 2.0 (Surflex-Dock)	−	DPP-4 Inhibition Assay; in vivo HFSD mouse model	[83]
Emodin and synthetic derivatives	*Cassia multi-juga* *Rumex japonicus* *Aloe vera*	5T4F	Molegro Virtual Docker 6.0	−	DPP-4 Inhibition Assay	[84]
Eleutherol Eleutherinoside A Eleuthoside B	*Eleutherine Bulbosa*	3KWF	AutoDock Vina 1.2.3 ver. 2020.	−	-	[85]
Caffeoylquinic acid derivatives Feruloylquinic acid derivatives Coumaroylquinic acid derivatives Protocatechuic acid derivatives	*Brassica oleracea*	1NU6	AutoDock Vina 4.2	−	-	[68]

‘+’ indicates that MD simulation was performed; ‘−’ indicates that MD simulation was not performed.

**Table 7:** Saponins docked to DPP-4.

Compound	Reported Natural Source	PDB ID	Docking Software	MD	Experimental Validation	Reference
Borassoside D Borassoside E Protodioscin Pennogenin triglycoside Pennogenin tetraglycoside	*Trillium govanianum*	5Y7J	Glide	−	DPP-4 Inhibition Assay	[86]
Gymnemasaponin II Gymnemaside II Gymnemic acid I Deacylgymnemic acid	*Gymnema sylvestre*	-	CB-Dock	−	-	[87]
Calenduloside E	*Allium sativum*	6B1E 4FFW 3WQH	Flare (Cresset)	−	DPP-4 Inhibition Assay, in vitro	[88]
Polygosides A	*Polygonatum odoratum*	3VJK	AutoDock Vina 1.1.2	+	-	[89]
Ginsenoside Rg5	*Panax ginseng*	3VJK	AutoDock Vina 1.1.2	+	-	[89]

‘+’ indicates that MD simulation was performed; ‘−’ indicates that MD simulation was not performed.

**Table 8:** Sterols docked to DPP-4.

Compound	Reported Natural Source	PDB ID	Docking Software	MD	Experimental Validation	Reference
Stigmasterol β-sitosterol	*Urena lobata*	-	dockingserver.com	−	DPP-4 Inhibition Assay	[35]
Stigmasterol β-sitosterol	*Pueraria tuberosa*	4FFV	YASARA	−	DPP-4 Inhibition Assay; in vivo	[36]
Stigmasterol α-spinasterol	*Abelmoschus manihot*	4FFW	AutoDock Vina PyRx 9.5	+	DPP-4 Inhibition Assay	[90]
Stigmasterol β-sitosterol	*Morinda citrifolia*	4PNZ	AutoDock	−	-	[91]
Campesterol β-sitosterol	*Trigonella foenum-graecum*	1X70	AutoDock Vina 1.1.2	+	-	[92]
Campesterol Stigmasterol β-sitosterol	Not reported	2ONC	AutoDock Vina	−	DPP-4 fluorometric assay	[93]
Campesterol Lanosterol Stigmasterol β-Sitosterol	*Glycine max*	Homology model (Swiss-Model; UniProt P27487)	dockingserver.com	−	-	[94]
Campesterol	*Allium cepa* *Helianthus annuus* *Luffa cylindrica* *Moringa oleifera*	2ONC	AutoDock 4.2 and AutoDock Vina	+	-	[22]

‘+’ indicates that MD simulation was performed; ‘−’ indicates that MD simulation was not performed.

**Table 9:** Terpenoids docked to DPP-4.

Compound	Reported Natural Source	PDB ID	Docking Software	MD	Experimental Validation	Reference
Linalool Myrcenol α-Elemol	*Cymbopogon citratus*	1X70	FRED and HYBRID	−	In vivo antidiabetic study	[95]
Valeranone Valencene Germacrene	*Amberboa ramosa*	2ONC	XP docking (Schrödinger suite)	+	DPP-4 Inhibition Assay; Kinetics studies	[96]
Caryophyllene oxide Myristicin Torreyol β-Elemene	*Oliveria decumbens*	5T4B	MOE Software	−	-	[97]
Citronellyl butyrate Citronellol Citronellyl formate Isomenthone Linalool α-Terpineol	*Plectranthus neochilus*	1WCY	AutoDock Vina tool from PyRx	−	-	[98]
Rebaudioside A Stevioside	*Stevia* sp.	3F8S	Molecular Docking Module using software Hex4e	+	-	[99]
Carnosol	*Rosmarinus officinalis*	1X70	AutoDock Vina	−	DPP-4 Inhibition Assay	[100]
Arjunic acid Arjunone Arjungenin Arjunetin	*Terminalia arjuna*	2QT9	Hex software 8.0.0	−	DPP-4 Inhibition Assay, in vivo STZ rat model	[101]
Thymoquinone	*Nigella sativa*	4A5S	AutoDock Vina	−	In vivo T2DM model; serum DPP-4 quantification (ELISA)	[102]
Ficusonolide	*Ficus foveolata*	2G63	Molecular Operating Environment (MOE 2016)	−	In vitro glucose uptake assay (L-6 cells) In vivo STZ-nicotinamide rat model	[103]
6′-O-Lactoyl Borapetoside B Borapetoside E Borapetoside F Rumphioside C Rumphioside I	*Tinospora crispa*	3G0B	Molegro Virtual Docker	−	-	[104]
β-Boswellic acid β-Keto-boswellic acid	*Boswellia* sp.	6B1E	Inverse docking approach MOE (v2014.09)	−	In vivo antidiabetic evaluation	[105]
24-methylcycloartanon Cycloartenon Cycloartenol Cycloeucalenol	*Artocarpus champeden*	1X70	AutoDock 4.2.6	−	-	[106]
Betulin	*Striga orobanchioides*	-	AutoDockVina 1.1.2	−	In vivo STZ-diabetic rat model	[107]
α-caryophyllene Pinene Ocimene	*Spilanthes filicaulis*	5Y7K	AutoDock Vina in PyRx 0.8	+	DPP-4 Inhibition Assay	[108]
6-α-hydroxyneopulchellin Ailanquassin Asiatic acid Aucubin Bigelovin Carnosol Jatropholone Rosmanol Tagitinin	Various plant-derived terpenoids (retrieved from the NPACT database)	3WQH	AutoDock Tools	+	-	[109]
CBD CBG CBN THC	*Cannabis* sp.	3BJM	AutoDock Vina	+	DPP-4 Inhibition Assay; Kinetic analysis (Lineweaver–Burk); Circular Dichroism	[110]
Salannin	*Chrozophora rottleri*	4A5S	AutoDock Vina	−	-	[46]
Friedelin	*Myrianthus serratus*	2P8S	AutoDock Vina 1.1.2	+	DPP-4 inhibition assay	[76]
α-Amyrin	*Elaeocarpus serratus*	4A5S	AutoDock software (v4.2.6)	−	-	[20]
Limonin	*Citrus aurantiifolia*	4A5S	AutoDock software (v4.2.6)	−	-	[20]
Cucurbitacin A, B	*Cucumis sativus*	4A5S	AutoDock software (v4.2.6)	−	-	[20]
Kuguacin B, H	*Momordica charantia*	6B1O	AutoDock Vina	−	In vivo (Wistar rats; RT-PCR gene expression)	[111]
Withanolide A Withacoagulin	*Withania coagulans*	2I03	Glide	−	-	[112]
Sitoindoside IX Withanoside IV	*Withania coagulans*	5Y7K	AutoDock 4.2	−	DPP-4 Inhibition Assay, HOMA analysis	[113]
Yuccagenin	*Curculigo orchioides*	2ONC	AutoDock 4.2 and AutoDock Vina	+	-	[22]
Glochidon	*Phyllanthus debilis*	2G63	AutoDock Vina	+	DPP-4 Inhibition Assay; In vivo OGTT	[114]
Eucalyptol α-Terpineol Borneol	*Aframomum melegueta*	5KBY	Glide	−	-	[115]
Withanolides (withanolide B,D,E, withaferin A, withanone, withangulatin A, withacoagulin H, sitoindoside IX)	*Withania coagulans*	5Y7K	AutoDock 4.2.6	−	DPP-4 Inhibition Assay; In vivo T2DM rat model	[113]
Coagulin L	*Withania coagulans*	6B1E	Glide, AutoDock 4.0	+	-	[116]
Auraptene Citral β-Bisabolene	*Aegle marmelos*	4N8E	AutoDock Vina	+	-	[117]
α-Humulene β-Caryophyllene β-Gurjunene β-Pinene Camphene Eucalyptol Myrcene Germacrene D Tagitinin A Tagitinin C Tagitinin F Tirotundin Tithonine Diversifolin	*Tithonia diversifolia*	4A5S	AutoDock Vina	−	-	[118]
Withacoagin Withanolide E	*Withania coagulans*	5Y7K	AutoDock	+	DPP-4 Inhibition Assay; In vivo STZ-nicotinamide T2DM rat model	[23]
Betulin Betulinic acid	*Ruellia tuberosa*	4A5S	Glide	−	-	[61]
3-oxo-α-ionol Loliolide	*Peronema canescens*	3G0B	Molegro Virtual Docker 5.0	−	DPP-4 Inhibition Assay	[63]
Kravanhin C	*Amomum tsao-ko*	2P8S	AutoDock 4.2.6	−	DPP-4 Inhibition Assay	[119]
Beta-amyron Lup-20(29)-en-3-one Lupeol Soyasapogenol B	*Dalbergia sissoo*	1X70	AutoDock Vina	+	-	[26]
6′-O-Lactoyl Borapetoside B Borapetoside C Borapetoside D Rumphioside B	*Tinospora crispa*	3G0B	Molegro Virtual Docker	−	DPP-4 Inhibition Assay	[120]

‘+’ indicates that MD simulation was performed; ‘−’ indicates that MD simulation was not performed.

**Table 10:** Levels of validation used in studies of natural DPP-4 inhibitors.

Evidence Level	Interpretation
Docking only	Low confidence
Docking + MD	Moderate confidence
Docking + DPP-4 inhibition assay	Moderate-to-high confidence
Docking + DPP-4 inhibition assay + cell studies	High confidence
Docking + DPP-4 inhibition assay + in vivo studies	High confidence
Docking + MD + comprehensive experimental validation	Highest confidence

## Data Availability

No new data were created or analyzed in this study. Data sharing is not applicable to this article.

## Supplementary Materials

The following supporting information can be downloaded at: https://www.mdpi.com/article/10.3390/pharmaceutics18060741/s1. Table S1: Peptides docked to DPP-4; Table S2: Detailed interaction profiles of natural compounds with DPP-4 residues.

## Abbreviations

The following abbreviations are used in this manuscript:

DPP-4	Dipeptidyl Peptidase-4
GLP-1	Glucagon-like Peptide-1
GIP	Glucose-dependent Insulinotropic Polypeptide
T2DM	Type 2 Diabetes Mellitus
MD	Molecular Dynamics
PDB	Protein Data Bank
