# Supplementary material for: Molecular Docking of Natural Compounds as DPP-4 Inhibitors in Type 2 Diabetes: A Comprehensive Review
Source: Pharmaceutics. 2026 Jun 15;18(6):741. doi: 10.3390/pharmaceutics18060741 (PMC13306514; doi:10.3390/pharmaceutics18060741)
Supplement: Supplementary file 1 [file pharmaceutics-18-00741-s001.zip › pharmaceutics-4362355-supplementary.pdf]

Supplementary materials

Table S1. Peptides docked to DPP-4

Peptide sequences	Natural source	PDB ID	Docking software	MD	Experimental validation	Reference
EGLELLLLLLAG AKSPLF FEELN TTNPLF TTGGKGGK ATNPLF QTPF LSKSVL LKEGGK SGPFGPK	*Phaseolus vulgaris*	3W2T	AutoDock	-	DPP-4 Inhibition Assay	[121]
Soy 1 (IAVPTGVA), Soy 2 (YVVNPDNDEN), Soy 3 (YVVNPDNNEN), Lup 1 (LTFPGSAED), Lup 2 (LILPKHSDAD), and Lup 3 (GQEQSHQDEGVIVR)	*Glycine max Lupinus spp.*	4PNZ	PLANTS	-	DPP-4 Inhibition Assay	[122]
IVVTRGRAT QEDDNRR RAPRMRWI REEEQQR QEERQEQR AGGEPRDGQSGQ MRPDEDEQEGQ DDEENPRDPRE GNPDDEFRPQ	*Juglans regia*	4PNZ	DOCK 6.9	-	DPP-4 Inhibition Assay	[123]
ASGLCPEEAVPRR	*Picrorhiza kurroa*	1X70	AutoDock Vina (v.1.5.6)	-	DPP-4 Inhibition Assay; in vitro	[124]
Sequences not individually specified	*Corylus heterophylla*	3WQH	CDOCKER, Autodock Vina and LeDock	+	-	[125]
LPFYPQ, GPVTPPILG, LPFYPQGV	*Sorghum bicolor*	1X70	AutoDock Tools. Vina1.1.2 s	-	DPP-4 Inhibition Assay, in vitro	[126]
IPQHY, VPQHY, VAVVPF, and VPLGGF	Oat proteins	2BGR	CDOCKER	-	DPP-4 Inhibition Assay	[127]
DGLGYY, AALWE, MFTGPY, YDLHGY, GESWCR, CCGDYY, NGENDWR, TWVV, GSYHDSK,	*Erythrina edulis*	1NU8	YASARA	+	In vitro	[128]

YYLTR, SQLPGW, GPPW, YPSY, SKDAPY						
AAWPGHPEF LAFP IAIPPGIPYW PPGIPYW	*Cicer arietinum*	5J3J	AutoDockTools 1.5.6	-	In vitro	[129]
LPQNIPPL, YPYY, YPW, LPYPY, WWW, YPY, YPF, WS, WYR, FPGPIPN, WWK, WFR, HRW, YWK, FWR, HNW	*Cannabis sativa*	3WQH, 4PNZ, 5YP3, 1NU8, 4J3J	Autodock 4.0	-	In vitro	[130]
IPPG<br>SPH	*Chenopodium quinoa*	1X70	AutoDock Tools software (version 1.5.6)	-	In vitro	[131]
HF, IW, YF,QF,YL, FR, KF, VF,YA, HL,NR,PH	*Cicer arietinum*	4PNZ	DockingRMSD platform, AutoDock Vina 1.1.2	-	-	[132]
AVPFWM, YSGWLGL, AHAGFGMLY	*Lupinus mutabilis*	1X70	The AutoDock Vina	-	DPP-4 Inhibition Assay, in vitro	[133]
ATFFPQ; TFFPQ; LPFYPN.	*Coix lacryma-jobi*	1X70	AutoDock Vina 1.1.2	-	DPP-4 Inhibition Assay	[134]
KGHLFPN	*Juglans mandshurica*	5T4B	Discovery Studio 2017 R2 software (Biovia)	-	-	[135]
PSF, IPG, SPR, CSPG, PPN, SPF	*Chenopodium quinoa*	1X70	The AutoDockTools2	-	-	[136]
LLPSY, NAPALVY	*Allium tuberosum*	1WCY	CDOCKER	-	DPP-4 Inhibition Assay	[137]
FNE, PM, SNLNFF	*Vigna angularis*	1RWQ	The AutoDock Tools	-	DPP-4 Inhibition Assay; in vitro	[138]
EPWWPK, LLTPKF, NLLMPH	*Vigna subterranea*	1NU6	pyDockEneRes server	-	DPP-4 Inhibition Assay	[139]
LFF, IGF, IYF	*Hippophae rhamnoides*	5J3J	AutoDockTools-1.5.7	-	DPP-4 Inhibition Assay	[140]
IPI KFPF IFR KGFL KIPF KLF	*Juglans regia*	5J3J	AutoDock software	-		[141]

					DPP-4 Inhibition Assay;<br><br>Stability tests	
LPGF, MPLPA, LPGFF	*Cucurbita moschata*	1WCY	AutoDock Vina (version 1.1.2)	+	DPP-4 Inhibition Assay<br><br>Stability tests	[142]
VPL, LGG, VPG, GPL	*Parkia timoriana*	1WCY	Not specified (CHARMm used for energy minimization)	-	DPP-4 Inhibition Assay	[143]
FPQPQ<br>FPRPF<br>YGGWN	Highland barley (*Hordeum vulgare*)	5J3J	AutoDock Tools 1.5.6.	+	DPP-4 Inhibition Assay; in vitro	[144]
KALVAP WPLVAP WALVAP YALVAP YPLVAP VPLVAP VALVAP KPLVAP IPLVAP IALVAP	*Ziziphus jujuba*	4A5S	Discovery Studio 2020	+	DPP-4 Inhibition Assay; in vitro	[145]
IPYWTY, IPYWT, LPNYN, LAFPGSS	*Pisum sativum*	1WCY	Auto Dock Vina	-	DPP-4 Inhibition Assay; in vitro	[146]
LSICGEESFGTGSDHIR<br>SLGESLLQEDVEAHK<br>QLRDIVDK	*Sorghum bicolor*	1NU8	ICM-Pro 3.8-4a (Molsoft)	-	DPP-4 Inhibition Assay; in vitro (Caco2)	[147]

Table S2. Detailed interaction profiles of natural compounds with DPP-4 residues

Interaction types are reported as described in the original publications; where not specified, only interacting residues are listed.

**Alkaloids**			
**Compound**	**Reported natural source**	**Reported interactions and residues**	**Reference**
Glycosin	*Rhizophora apiculata*	Arg, Trp, Tyr, Thr, Glu, His, Ser	[15]
Berberine	*Cardiospermum halicacabum*	π–anion (Glu206), π–π stacking (Tyr666)	[16]
Palmatine	*Fibraurea tinctoria*	Tyr547, Tyr585, Lys554, Cys551	[17]
Berberine	*Fibraurea tinctoria*	Tyr547, Tyr585, Tyr662, Gln553	[17]
Tetrandrine	*Phaeanthus ophthalmicus*	H-bond (Ala133, Lys226); hydrophobic (Leu130, Glu134); π–amide (Arg222); alkyl (Ala223, Arg219)	[18]
Limacusine	*Phaeanthus ophthalmicus*	H-bond (Pro178); electrostatic (Asp180, Arg240); hydrophobic (Arg240)	[18]
Colchicine	*Schleichera oleosa*	Lys554, Tyr547, Asn710, Tyr662, Arg125, His740, Ser630, Trp629	[19]
Anonaine	*Annona squamosa*	Glu205, Glu206, Phe357, Tyr666, Arg669	[20]
Elaeocarpidine	*Elaeocarpus serratus*	Arg125, Asp545, Val656, Lys554, Trp563, Asn562, Ser630, Asn710, His740	[20]
Elaeocarpine	*Elaeocarpus serratus*	Lys122, Trp124, Trp201, Phe240, Lys250, Val254	[20]
Actinodaphnine	*Litsea glutinosa*	Glu205, Glu206, Tyr662, Ser630	[21]
Coreximine	*Litsea glutinosa*	Glu205, Glu206, Ser209, Arg358	[21]
Withasomnine	*Withania coagulans*	Glu206	[23]
Berberine	*Coptis chinensis*	Ser630, Arg669; hydrophobic contacts with Glu205, Glu206	[24]
Lauroscholtzine	*Dalbergia sissoo*	H-bond (Gly741, Tyr752, Asp545)	[26]
Corydine	*Dalbergia sissoo*	H-bond (Lys554, Val546, Asp545)	[26]

**Coumarins**			
**Compound**	**Natural source**	**Reported interactions and residues**	**Reference**
Coumarin	Not reported	H-bond (Gln731, Ala732)	[28]

Coumarin	*Cinnamomum burmannii*<br>*Caesalpinia sappan*	Glu206, Val207, Ser209, Phe357, Arg358, Arg125, Tyr547, Ser630, Tyr631, Tyr662, Val656, Trp659, Tyr666, Asn710, Val711, His740	[29]
Scopoletin	*Lunasia amara*	Carbon–H bond; van der Waals; π–anion; π–alkyl; π–sigma	[30]
Dicoumarol	*Schleichera oleosa*	Ser209, Glu205, Asn710, Arg125, His740, Ser630, Val711, Tyr666, Val656, Tyr662, Tyr631, Tyr547, Glu206, Arg669, Phe357	[19]
Cichoriin	*Fraxinus hupehensis*<br>*Calea fruticosa*	Tyr547, Ser630, Ser209, Phe357, Glu206, Tyr662	[31]

**Flavonoids**			
**Compound**	**Reported natural source**	**Reported interactions and residues**	**Reference**
Hesperetin	*Citrus spp*. and berries	Arg358, Arg669, Glu206	[34]
Naringenin	*Citrus spp*. and berries	Arg356, Arg358, Glu361, Ser209	[34]
Genistein	*Citrus spp*. and berries	Arg356, Phe357, Arg358, Glu206	[34]
Apigenin	*Citrus spp*. and berries	Arg356, Arg358, Glu205, Ser209	[34]
Kaempferol	*Citrus spp*. and berries	Ser209, Arg356, Phe357, Arg358, Glu361	[34]
Quercetin	*Citrus spp*. and berries	Arg356, Arg358, Glu206, Ser209	[34]
Kaempferol glycosides	*Lens culinaris*	Glu205, Glu206, Gln533, Arg560, Gly741, Tyr752; Tyr547 (water-mediated)	[37]
Quercetin	Not reported	Val738, Ser720, Tyr700, Ala732, Met733	[28]
Isorhamnetin glycosides	Not reported	Trp124, Arg125, Glu204, Trp629, Ala654, Asp739, Gly741, Leu598, Pro655, Arg658, Tyr661, Tyr683, Thr667	[38]
Cyanidin glycoside	Not reported	Pro655, Arg658, Glu660, Asp663, Tyr670, Tyr683, His712, Gln715	[38]
Rutin	Not reported	Phe357 (π), Tyr585, Ser630, Arg125, Tyr547, Tyr662, Glu206, Arg669	[39]
Taxifolin	Not reported	Tyr547, Glu206, Arg669	[40]
Rutin<br>Quercetin-3-O-β-glucoside	*Solanum elaeagnifolium*	Glu206, Tyr547, Tyr662, Asn710, Arg125, Arg669	[41]

Naringin<br>Kaempferol<br>Chalcone			

Phenolics (non-flavonoid)			
**Compound**	**Natural source**	**Reported interactions and residues**	**Reference**
Resveratrol	*Citrus spp.* and berries	Arg669, Ser630, Ser209, Glu206	[34]
Gallic acid	*Citrus spp.* and berries	Arg356, Phe357, Arg358, Tyr585, Ile405	[34]
Caffeic acid	*Citrus spp.* and berries	Arg358, Arg669, Glu206	[34]
Caffeic acid<br>Lauric acid<br>Capric acid<br>Caprylic acid	*Cocos nucifera*	Arg358	[69]
Gallic acid	*Cocos nucifera*	Trp215, Val303, Arg358	[69]
Oleuropein<br>Oleacein<br>Oleocanthal	*Olea europaea*	Trp629 (π–π), Tyr547 (π–π), Ser630, His740, Asn710, Phe357, Val546, Lys554	[70]
Gallic acid<br>p-Coumaric acid<br>Caffeic acid 4-O-glucoside<br>4-hydroxybenzaldehyde	*Bambusa arundinacea*<br>*Oryza sativa*	Glu205, Glu206, Arg358, Ser209, Tyr547, Ser630, Tyr666	[71]
Piceatannol<br>Resveratrol<br>Dihydropiceatannol<br>Chrysophanol<br>Emodin	*Senna siamea*	Glu205, Asp739, Gln123, Tyr238, Arg253, Asp192, Lys122, Asp737, Lys250, Tyr752, Asp545	[72]
Oleuropein	Not reported	Arg125, Tyr666, Tyr547, Phe357 (π), Arg669, Glu206	[39]
Calebin A	*Curcuma longa*	Glu206, Tyr547, Ser630, Tyr662, Asn710, Arg125, Arg669, Tyr585, Ser552, Cys551	[73]
Resveratrol	Not reported	Tyr547, Arg669, Glu206, Val207, Arg358	[40]
Peperochromene A	*Peperomia pellucida*	Phe357 (π–π), Tyr662, Tyr666	[74]

Saponins			
**Compound**	**Natural source**	**Reported interactions and residues**	**Reference**
Calenduloside E	*Allium sativum*	His741, Arg123, Tyr548, Tyr632, Phe355, Glu203, Arg670, Glu204, Ile205, Phe206, Arg356, Ser631, Gly633, Trp630, Tyr667	[88]

Sterols			
**Compound**	**Natural source**	**Key interactions and residues**	**Reference**
α-spinasterol	*Abelmoschus manihot*	van der Waals: Arg123, Glu203, Glu204, Ile205, Gly207, Arg356, Ser631, Arg670, Asn711; π–σ: Phe355; π–alkyl: Tyr548, Tyr632, Val657, Tyr663, Tyr667, Val712	[90]
stigmasterol	*Abelmoschus manihot*	van der Waals: Arg123, Glu203, Glu204, Ile205, Gly207, Tyr548, Ser631, Tyr632, Val657, Trp660, Asn711; π–σ: Phe355; π–alkyl: Tyr663, Tyr667	[90]
β -sitosterol	*Morinda citrifolia*	Val207, Arg358	[91]
stigmasterol	*Morinda citrifolia*	Val207	[91]
β sitosterol	*Trigonella foenum-graecum*	Trp629, Val546, Arg125, Asp739, Lys122, Gly741, Asp545, Ser630, Tyr547, Gly628, Lys554	[92]
Campesterol	*Trigonella foenum-graecum*	Val711, Ser630, Tyr631, Tyr662, Glu205, Arg358, Ser209, Glu206, Phe357, Tyr666, Tyr547	[92]

Terpenoids			
**Compound**	**Natural source**	**Reported interactions and residues**	**Reference**
Linalool	*Cymbopogon citratus*	Tyr662A	[95]
Myrcenol	*Cymbopogon citratus*	Glu205A	[95]
α-Elemol	*Cymbopogon citratus*	Tyr662A, Glu205A	[95]
Citronellyl butyrate, Citronellol, Citronellyl formate, Linalool, α-Terpineol, Isomenthone	*Plectranthus neochilus*	Asp709, Lys122, Phe240, Ala707, Val252	[98]
